# An *in situ*-Synthesized Gene Chip for the Detection of Food-Borne Pathogens on Fresh-Cut Cantaloupe and Lettuce

**DOI:** 10.3389/fmicb.2019.03089

**Published:** 2020-02-05

**Authors:** Sarengaowa, Wenzhong Hu, Ke Feng, Aili Jiang, Zhilong Xiu, Ying Lao, Yuanzheng Li, Ya Long

**Affiliations:** ^1^School of Bioengineering, Dalian University of Technology, Dalian, China; ^2^College of Life Science, Dalian Minzu University, Dalian, China; ^3^Key Laboratory of Biotechnology and Bioresources Utilization, Ministry of Education, Dalian, China

**Keywords:** *Salmonella* Typhimurium, *Vibrio parahemolyticus*, *Staphylococcus aureus*, *Listeria monocytogenes*, *Escherichia coli* O157:H7, *in situ*-synthesized gene chip, tiling array, fresh-cut fruits and vegetables

## Abstract

Fresh foods are vulnerable to foodborne pathogens which cause foodborne illness and endanger people’s life and safety. The rapid detection of foodborne pathogens is crucial for food safety surveillance. An *in situ*-synthesized gene chip for the detection of foodborne pathogens on fresh-cut fruits and vegetables was developed. The target genes were identified and screened by comparing the specific sequences of *Salmonella* Typhimurium, *Vibrio parahemolyticus*, *Staphylococcus aureus*, *Listeria monocytogenes*, and *Escherichia coli* O157:H7 from the National Center for Biotechnology Information database. Tiling array probes were designed to target selected genes in an optimized hybridization system. A total of 141 specific probes were selected from 3,227 hybridization probes, comprising 26 *L. monocytogenes*, 24 *S. aureus*, 25 *E. coli* O157:H7, 20 *Salmonella* Typhimurium, and 46 *V. parahemolyticus* probes that are unique to this study. The optimized assay had strong amplification signals and high accuracy. The detection limit for the five target pathogens on fresh-cut cantaloupe and lettuce was approximately 3 log cfu/g without culturing and with a detection time of 24 h. The detection technology established in this study can rapidly detect and monitor the foodborne pathogens on fresh-cut fruits and vegetables throughout the logistical distribution chain, i.e., processing, cleaning, fresh-cutting, packaging, storage, transport, and sale, and represents a valuable technology that support the safety of fresh agricultural products.

## Introduction

The incidence of foodborne diseases resulting from the consumption of food contaminated with pathogens has increased significantly in many countries over the last few decades (Fusco and Quero, 2014). In 2011, 69,553 cases of food poisoning resulted in 7,125 hospitalizations and 93 deaths in Europe (European Food Safety Autority [EFSA] and European Centre for Disease Control, and Prevention [ECDC], 2013). The total cases from foodborne diseases reach 48,000,000 individuals, with 128,000 patients in hospitals, and cause 3,000 deaths each year in the United States according to the report from the Centers for Disease Control and Prevention (CDC) (Center for Disease Control and Prevention [CDC], 2011; Scallan et al., 2011). Food poisoning from fresh agricultural products contaminated with pathogens also occurs with surprising frequency. In the United States, the outbreak of foodborne illness was related to cantaloupe, which infected 1,751 individuals and caused 34 deaths (Michelle et al., 2014; Feng et al., 2016). With the increase in the demands for nutritious, healthy, and convenient foods, the consumption of fresh-cut produce involving fresh agricultural products has increased substantially worldwide (Oliveira et al., 2015; Ma et al., 2017). However, the processing of fresh-cut produce by cutting, shucking, carving, slicing, and peeling is highly susceptible to contamination by microorganisms and may lead to leakage or loss of nutrients (Gleeson and Beirne, 2005).

A potential safety risk from the contamination of foodborne pathogens from seed, soil, irrigation water, and organic fertilizers exists in fresh-cut fruit and vegetable products (Hoagland et al., 2018). Some studies have shown that some foodborne pathogens can survive and reach a high density (∼6 log cfu/g) on fresh-cut melon, papaya, celery, onions, and tomatoes (Penteado and Leitão, 2004; Feng et al., 2015; Salazar et al., 2017; Jayeol et al., 2019). Disease outbreaks were associated with foodborne pathogens on fresh-cut produce (Alegre et al., 2010; Center for Disease Control and Prevention [CDC], 2013). For example, fresh-cut cantaloupe, watermelon, and honeydew contaminated with *Salmonella* Carrau and *Salmonella* Adelaide resulted in the hospitalization of 38 and 36 people in 2018 and 2019, respectively (Center for Disease Control and Prevention [CDC], 2018, 2019).

The traditional pathogen detection methods are excessively time-consuming, which takes up to 8 days to obtain the results, making these methods inadequate for the rapid identification of foodborne pathogens (Lazcka et al., 2007). Considering that the shelf-life of fresh-cut fruits and vegetables ranges from 3 to 5 days, more rapid pathogen detection methods are required for these products. Detection methods employing non-culture-based identification and quantification technologies, such as real-time PCR and quantitative reverse-transcription PCR, are less time-consuming than the traditional methods but are susceptible to amplification biases and errors (Wolffs et al., 2005). For example, false-positive amplification can occur among genetically related microorganisms in the environment, and false-negative amplification can result from sequence variation in the primer binding sites of the target diagnostic region or from low reaction sensitivity (Davison, 1999).

To overcome these limitations, high-throughput DNA microarrays that can rapidly identify foodborne pathogens with a high degree of specificity have been developed (Kostić and Sessitsch, 2012). The DNA microarrays comprise of hundreds of oligonucleotide probes to positively detect a single target pathogen. Arraying many specific probes and setting a sufficiently high threshold for the positive identification of the presence of a target pathogen substantially avoid the false-positives that result from cross-contamination between foodborne pathogens (Wilson et al., 2002). The traditional gene chip arrays contain some probes that target the coding sequence of the virulence gene of the target pathogens (Mockler and Ecker, 2005; Bertone et al., 2005). The identification for foodborne pathogens is no longer considered reliable when only one area of the genome is targeted. The strategy of tiling probe arrays as applied on the gene chip can target the contiguous genome regions of the target foodborne pathogen and detect the base of the target gene sequences (Selinger et al., 2000; Johnson et al., 2005). For the development of a microarray detection technology, no such chip is commercially available, and the tiling arrays must be designed and synthesized for specific purposes. The customization of arrays permits total control over the probes on the chip, allowing researchers to select specific probes for the detection of the pathogen and to control the distribution of probes over the array. The *in situ*-synthesized gene chips are high-density arrays that have high specificity and can rapidly screen and identify the sources of contamination and the foodborne pathogens during food poisoning outbreaks.

The purpose of this research was to develop a rapid detection technique based on an *in situ*-synthesized gene chip comprising of virulence genes for detecting *Salmonella* Typhimurium (ST), *Listeria monocytogenes* (LM), *Staphylococcus aureus* (SA), *Vibrio parahemolyticus* (VP), and *Escherichia coli* O157:H7 (EC O157:H7), which are commonly associated with fresh-cut fruits and vegetables. The pathogens targeted by the chip (LM, ST, SA, EC O157:H7, and VP) are clinically relevant to food safety. The tiling probes of the whole target gene of the pathogens on the microarrays were used to enhance the detection accuracy of the chip. The *in situ*-synthesized virulence gene array can be used as a diagnostic tool in food poisoning outbreaks related to fresh-cut fruits and vegetables contaminated with foodborne pathogens.

## Materials and Methods

### Fresh-Cut Cantaloupe and Lettuce

Fresh cantaloupes and lettuces were purchased from the New-Mart supermarket in Dalian City, China. They were chosen based on uniformity of maturation stage, size, and absence of defects or injuries. The samples were stored at 4°C for approximately 1 h (transport time) prior to processing. The fresh cantaloupe and lettuce were cleaned twice with 75% (*v*/*v*) distilled water, sterile water, and ethyl alcohol (Tianjin Kemiou Chemical Reagent Co., Ltd., Tianjin, China), respectively. The samples were dried in a biosafety cabinet (1300 Series A2 Class II, Type A2, Thermo Fisher Scientific, Shanghai, China) prior to cutting. The cantaloupe was peeled and cut into 1-cm^3^ cubes without a rough outside surface. The lettuce was cut into 1-cm^2^ pieces using a sterile knife.

### Bacterial Strains

The bacterial strains including ST (CICC 21484), LM (CICC 21633), SA (CICC 21600), VP (CICC 21617), and EC O157:H7 (CICC 21530) were purchased from the China Center of Industrial Culture Collection (CICC, Beijing, China). The LM was preserved in trypticase soy–yeast extract broth (Qingdao Hopebio-Technology Co., Ltd., Qingdao, Shandong, China) with 80% glycerol (Tianjin Kemiou Chemical Reagent Co.) at −20°C. The SA, EC O157:H7, and ST were preserved in TSB (Qingdao Hopebio-Technology Co.) with 80% glycerol at −20°C. The VP was stored at −20°C in 3% NaCl alkaline peptone water (Qingdao Hopebio-Technology Co.) with 80% glycerol. All bacterial strains were revived from frozen stocks before use by cultivation in a culture medium at 37°C for 24 h. The bacterial suspensions were made using 0.1% (*w*/*v*) peptone water (Aobox Biotechnology, Beijing, China) to obtain the proper concentration. The LM was enumerated on trypticase soy agar (TSA) with 0.6% (*w*/*v*) yeast extract (Qingdao Hopebio-Technology Co.). The ST, SA, and EC O157:H7 were enumerated on TSA (Qingdao Hopebio-Technology Co., Ltd.). The VP was enumerated on 3% NaCl TSA (Qingdao Hopebio-Technology Co., Ltd.). All bacteria were cultured for 24 h at 37°C, and the colonies were measured with an aCOLyte colony counter (Acolyte Technologies Corp., London, United Kingdom). The microbial counts are expressed as log cfu/ml.

### DNA Extraction

For DNA extraction, the bacterial suspensions (1 ml of 10^8^ cfu/ml) were centrifuged at 12,000 × *g* for 2 min. The sediment was suspended and washed twice using 0.1% peptone water. Then, the samples were centrifuged again at 12,000 × *g* for 2 min. The genomic DNA was obtained from foodborne pathogens according to the following procedure of DNA extraction: add 200 μl of cetyltriethylammnonium bromide extraction buffer (Beijing Solarbio Science and Technology Co., Ltd., Beijing, China) to mix and thoroughly vortex the sediment of 1 ml of suspension, transfer the homogenate to a 65°C bath for 30 min, place the sample at −80°C for 15 min, and put it at 65°C for 30 min again; repeat this process once for freezing and thawing, add 200 μl of chloroform/isoamyl alcohol (24:1), vortex for 5 s, and then centrifuge the sample at 12,000 × *g* for 15 min to separate the phases; transfer the upper aqueous phase to a new tube containing 80 μl magnetic beads (DNA Binding Beads, Thermo Fisher Scientific, MA, United States), incubate the sample at room temperature (∼25°C) for 5 min to allow the DNA to bind to the beads, and place the sample on the magnetic separator (24-well magnetic separator, Thermo Fisher Scientific) for 5 min. Without removing the plate from the magnetic separator, carefully remove and discard the supernatant, subsequently wash it with 80% ethanol twice, remove the residual ethanol by drying in a biosafety cabinet, and then dissolve DNA in 20 μl TE buffer (TE Buffer, Thermo Fisher Scientific). The genomic DNA concentration was determined using a Multiskan FC Microplate Photometer (Thermo Fisher Scientific), and the purity was calculated by the ratio between the OD of the DNA at 260 nm and at 280 nm.

### Target Gene Screening

The entire genome sequences of the five foodborne pathogens were obtained from the GeneBank sequence database produced by the National Center for Biotechnology Information (NCBI). The number of genome sequences for the LM, ST, SA, VP, and EC O157:H7 was 31, 153, 93, 19, and 8, respectively, in all genomes (Supplementary Table S1). The sequences were initially screened using the NCBI BLAST to identify those with less than 80% sequence identity among the target strains and which were longer than 500 bp. Pairwise comparisons of the selected genome sequences were performed to identify similar sequences between any two genomes. For each bacterial genome, an inverse complement of the sequence operation was performed according to the sequence intervals of the obtained similarity comparison results to obtain the sequence intervals specific to the bacterial species. These sequences were compared against the NCBI database to screen out specific gene sequences and then aligned within the species to identify the conserved regions. Based on the specific sequences above, the target specific gene was screened for the second time according to the principle of sequence identity >95% intraspecific and <75% interspecific. The primers were designed based on the screening target gene of the LM, SA, EC O157:H7, ST, and VP (Table 1).

**TABLE 1:** Primer sequences.

**Strain**	**Gene**	**Primer sequences (5′–3′)**	**Length**
*Salmonella* Typhimurium	*inv*A	F: GTCCAGCTCTGTCGCCTTAATC R: ACGGCTCCCTGCTACGCT	932
*S. aureus*	*sasH*	F: TCTGATTCAATTCGTGTTTACTATGA R: CGCTACCTTTAGGCACTGACA	547
*E. coli* O157:H7	*wzy*	F: TATTTGATGATGATCCAGGGG R: ATTTTGTTCTCCGTCTTGTCCTA	350
*L. monocytogenes*	*hly*A	F: GCTCAAGCC(T)TATCCAAATGTAAGT R: CCCAGATGGAGATATTTCTATTTTTC	958
*V. parahemolyticus*	*comEC/rec2*	F: ACTAAGTTGGTCGGTTCGATATG R: TCTGGCTGCTTAGTTTGTGTTTAG	440

### Multiplex PCR

The optimized multiplex PCR condition contained 0.8 ng/μl of genomic DNA, 1 × ex Taq Buffer plus Mg^2+^, 0.2 mM of dNTPs, 2.5 U of ex Taq DNA polymerase, and 1 nM of Cy3-dCTP RNase-free water to a final volume of 50 μl. The primer concentrations were 0.46 μM for LM, SA, and VP, 0.44 μM for ST, and 0.17 μM for EC O157:H7. All primers and reagents in the multiplex PCR assay were obtained from Takara Bio. Inc. (Dalian, Liaoning, China). The thermal profile for the assay includes incubating for 2 min at 95°C for pre-degeneration, subsequently setting 35 cycles involving 30 s at 95°C for denaturing, 30 s at 55°C for annealing, and 30 s at 72°C for extension, followed by 10 min at 72°C for the final extension. The amplicon of multiplex PCR was separated by electrophoresis on 3% agarose gels supplemented with Gelred dye (Biotium, Inc., Hayward, CA, United States) and visualized on a UVP BioSpectrum Imaging System (UVP, LLC, CA, United States). The multiplex PCR products were purified with LCS beads (LC science, Houston, TX, United States) to remove the redundant primers and the impurities.

### Design of Tiling Probe Arrays

A microfluidic chip made by *in situ*-synthesis was manufactured by LC Sciences (Houston, TX, United States). The chip contains 3,968 probe sites (128 rows × 31 lines) in 1.4 cm^2^. The tiling array probes (25 bp in length) were designed according to the lengths and the region of the PCR amplicons derived from LM, SA, EC O157:H7, ST, and VP. The adjacent probe sequences were separated by a single base until the entire target sequence of each of the five target strains was covered. A total of 3,227 probes were obtained: 958 from LM, 547 from SA, 350 from EC O157:H7, 932 from ST, and 440 from VP. The layout of the tiling array probes is shown in Supplementary Table S2.

### Microarray Hybridization, Washing, and Scanning

The preparation of chips for hybridization, target hybridization, washing, and scanning were performed by the procedure described as follows (LC Science, Houston, TX, United States). Briefly, the mixture of 25 μl of PCR amplification products (200 ng/μl) and 25 μl of hybridization buffer was added on the *in situ-*synthesis gene chip. Subsequently, hybridization for 18 h at 40°C, washing at 40°C, and staining were performed in a fluidics station at LC Science. The hybridization buffer (1 ml) was flushed through the chip at 500 μl/min for 20 min. The array chips were scanned using a GenPix 4000B scanner (Molecular Devices/Axon, Sunnyvale, CA, United States). The scanning pixel size was 10 mm. The Cy3 signals were collected using 532-nm channels (Zhu et al., 2007).

### Internal Verification and Optimized Probe Specificity

To test the specificity among the target pathogens, the genomic DNA of each target pathogen (1 ml of 10^8^ cfu/ml) was extracted, and 0.8 ng/μl of genomic DNA was amplified in the PCR system (50 μl) as described above. The mixture (50 μl), with an equal ratio of PCR amplicons (200 ng/μl) and hybridization buffer, was hybridized to the constructed gene chip (as described above). The 100 probes with the strongest hybridization signals were selected for further analysis, and the probes that detect hybridization signals from the non-target bacteria were excluded. The array layout was repeated ten times to verify the accuracy of hybridization (Supplementary Table S3).

### Pathogens in Artificially Contaminated Fresh-Cut Cantaloupe and Lettuce

Fresh-cut cantaloupes (10 g) and lettuce (10 g) were placed on petri dishes and inoculated with 0.5 ml of the mixture of LM, SA, EC O157:H7, ST, and VP (each 0.1 ml of 3–4 log cfu/ml) using a sterile micropipette. Each sample was dried in a biosafety cabinet for 1 h and then put into a sterile stomacher bag (Interscience, Saint Nom la Breteche, France) containing 90 ml of 0.1% peptone water. The sterile stomacher bag was blended in a stomacher at high-speed setting for 1 min. The sample residues were removed using a nylon membrane with 15-μm pore in the filtration apparatus (Hangzhou Hengqing Technology Co., Ltd., Hangzhou, Zhejiang, China). The filtrate was collected again using a polyethersulfone filtration membrane with 0.22-μm pore. The filtration membrane enriching the foodborne pathogens was transferred to centrifuge tubes containing 10 ml of phosphate buffer (0.2 mM) and vortexed for 15 min. The genomic DNA of the bacterial suspensions was extracted as described above (“DNA Extraction”). The number of ST, LM, EC O157:H7, SA, and VP on fresh-cut cantaloupes was respectively, measured on *Salmonella* spp. chromogenic media and Oxford agar base (both from Qingdao Hopebio-Technology), EC O157:H7 chromogenic media, SA chromogenic media, and VP chromogenic media (from Shanghai Central Bio-Engineering Ltd., Co., Shanghai, China). The array layout was repeated 12 times on the *in situ*-synthesized gene chip (Supplementary Table S4). The multiplex PCR amplification products from the genomic DNA of the target pathogens hybridized with probes and hybridization signals on the *in situ*-synthesized gene chip were detected as described above (“Microarray Hybridization, Washing, and Scanning”).

### Data Analysis

The fluorescence signal values were extracted from the images using Array-Pro Analyzer software (Media Cybernetics Inc., Rockville, MD, United States), which yielded values between 0 and 65,535 arbitrary units. The positive results were defined as the difference value between the medians of the feature pixel intensities, and the background pixel intensities were above three times than the standard deviation of the background pixel intensities at 532 nm. The subsequent data were measured using Microsoft Excel (Microsoft Corporation, Redmond, WA, United States).

## Results

### Probe Screening for Detecting Pathogenic Bacteria

**FIGURE 1:**
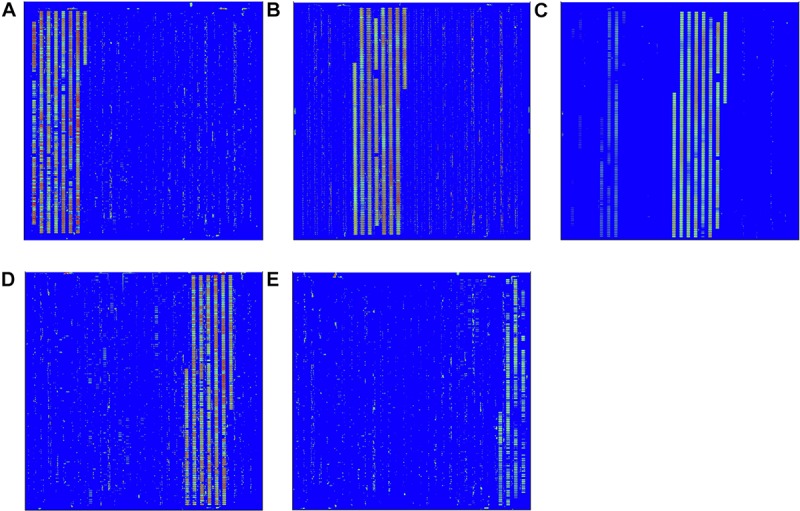
Probe screening on *in situ*-synthesized gene chip for detecting foodborne pathogens. **(A)** *Listeria monocytogenes*, **(B)** *Salmonella* Typhimurium, **(C)** *Staphylococcus aureus*, **(D)** *Vibrio parahemolyticus*, **(E)** *Escherichia coli* O157:H7. The array layout is presented as a list in Supplementary Table S2.

The PCR amplification product of each pathogen was respectively, hybridized with the *in situ*-synthesized gene chip containing the tiling array probes. The performance of the *in situ*-synthesized gene chip for the sequence-specific detection of amplification products of PCR was assessed (Figure 1). The fluorescence signal values were observed on the site which synthesized probes for detecting the target pathogen. The results of hybridization for LM, ST, SA, VP, and EC O157:H7 are shown in Figures 1A–E. The 100 probes with the strongest signal values were selected from the positive result of each target foodborne pathogen (Supplementary Tables S5–S9). However, some signal values were observed on the *in situ-*synthesized gene chip with non-target pathogen. It is obvious that the signal values were observed on the positive probe site of the LM from the *in situ*-synthesized gene chip with SA (Figure 1C). There are still some signal values on the other *in situ*-synthesized gene chips with non-target pathogen (Figures 1A,B,D,E). Therefore, the differences between the hybridization signal values of the top 100 probes and the non-target pathogenic bacteria was detected by Array-Pro software, and the probes with signal values similar to the negative probes were selected as final probe. A total of 141 specific probes were screened: 26 from LM, 20 from ST, 24 from SA, 46 from VP, and 25 from EC O157:H7 (Table 2).

**TABLE 2:** The specific probe for detection *Listeria monocytogenes*, *Salmonella* Typhimurium, *Staphylococcus aureus*, *Escherichia coli* O157:H7, and *Vibrio parahemolyticus.*

**Strain**	**Probe Sequence (5′–3′)**
*Escherichia coli* O157:H7	TTTACAACGATTGCTTTATTTGGTT
	CGTTCTGAATTGGTGTTGCTCATTC
	TTACAACGATTGCTTTATTTGGTTA
	CGATTGCTTTATTTGGTTATCGTTC
	CGTTCTGAATTGGTGTTGCTCATTA
	TATTTTACAACGATTGCTTTATTTG
	GTTATCGTTCTGAATTGGTGTTGCT
	AACGATTGCTTTATTTGGTTATCGT
	TTATCGTTCTGAATTGGTGTTGCTC
	GTTCTGAATTGGTGTTGCTCATTAT
	TATATTTTACAACGATTGCTTTATT
	ATATTTTACAACGATTGCTTTATTT
	GTTCTGAATTGGTGTTGCTCATTCT
	ACAACGATTGCTTTATTTGGTTATC
	GAGCATAAATTCAAACAGAGGACCA
	GAGCATAAATTCAAAAAGAGGACCA
	GCATTAATTATTCTTTATGATGAGC
	GGTTATCGTTCTGAATTGGTGTTGC
	TTTTGGTAATATAGTTGTGTTTGCA
	GGTGTTGCTCATTATTCAATATATA
	GGTGTTGCTCATTCTTCAATATATA
	GAATTGGTGTTGCTCATTATTCAAT
	GAATTGGTGTTGCTCATTCTTCAAT
	CATTAATTATTCTTTATGATGAGCA
	GAATAGCTGAAGGTAATGGACTTTA
*Listeria monocytogenes*	AAACTTCGGCGCAATCAGTGAAGGG
	GACCTTCCAGATTTTTCGGCAAAGC
	GCGCAATCAGTGAAGGGAAAATGCA
	TAAACTTCGGCGCAATCAGTGAAGG
	TTAATGAACCTACAAGACCTTCCAG
	GAATGTAAACTTCGGCGCAATCAGT
	TGTAAACTTCGGCGCAATCAGTGAA
	CAAGACCTTCCAGATTTTTCGGCAA
	ATGTTAATGAACCTACAAGACCTTC
	TAATGAACCTACAAGACCTTCCAGA
	AAAGCTGTTACTAAAGAGCAGTTGC
	TCGGCGCAATCAGTGAAGGGAAAAT
	GATTATGATGACGAAATGGCTTACA
	TGATGACGAAATGGCTTACAGTGAA
	ATGACGAAATGGCTTACAGTGAATC
	TCGGCAAAGCTGTTACTAAAGAGCA
	TTCCAGATTTTTCGGCAAAGCTGTT
	ATGATGACGAAATGGCTTACAGTGA
	TACAAGACCTTCCAGATTTTTCGGC
	GTGAAGGGAAAATGCAAGAAGAAGT
	CAAAGCTGTTACTAAAGAGCAGTTG
	CCTTCCAGATTTTTCGGCAAAGCTG
	AATGTTAATGAACCTACAAGACCTT
	TGAATGTTAATGAACCTACAAGACC
	GGGAAAATGCAAGAAGAAGTCATTA
	AGCTGTTACTAAAGAGCAGTTGCAA
*Vibrio parahemolyticus*	GTTACTCAAGTGTCGATACGATGAT
	GCTACTCAAGTGTCGATACGATGAT
	TACTCAAGTGTCGATACGATGATTT
	CTACTCAAGTGTCGATACGATGATT
	GGTTACTCAAGTGTCGATACGATGA
	GGCTACTCAAGTGTCGATACGATGA
	ACTCAAGTGTCGATACGATGATTTT
	TCAAGTGTCGATACGATGATTTTAA
	AGAGGTTACTCAAGTGTCGATACGA
	AGAGGCTACTCAAGTGTCGATACGA
	CAAGTGTCGATACGATGATTTTAAG
	AAGTCATGCTGATAATGACCATGCT
	AAGTCATGCCGATAATGACCATGCT
	TGTCGATACGATGATTTTAAGTCAT
	CGATACGATGATTTTAAGTCATGCC
	TCGATACGATGATTTTAAGTCATGC
	AGTCATGCTGATAATGACCATGCTG
	AGTCATGCCGATAATGACCATGCTG
	CGATACGATGATTTTAAGTCATGCT
	GATACGATGATTTTAAGTCATGCTG
	GATACGATGATTTTAAGTCATGCCG
	CGCAGAGGTTACTCAAGTGTCGATA
	CGCAGAGGCTACTCAAGTGTCGATA
	GTCATGCTGATAATGACCATGCTGG
	GTCATGCCGATAATGACCATGCTGG
	GAGGTTACTCAAGTGTCGATACGAT
	TAGCTGAGCAAGTGATTACGCCAGT
	GCTTGGCACAACGGCAGTATAGCTG
	GCTTGGCAAAACGGCAGTATAGCTG
	TCTATGATACGGGCAAGGCTTGGCA
	AAGTGTCGATACGATGATTTTAAGT
	GATTTTAAGTCATGCTGATAATGAC
	GATTTTAAGTCATGCCGATAATGAC
	GCAGAGGTTACTCAAGTGTCGATAC
	GCAGAGGCTACTCAAGTGTCGATAC
	CTCAAGTGTCGATACGATGATTTTA
	TACGATGATTTTAAGTCATGCTGAT
	TACGATGATTTTAAGTCATGCCGAT
	AGCTGAGCAAGTGATTACGCCAGTA
	TTAAGTCATGCCGATAATGACCATG
	ATACGATGATTTTAAGTCATGCCGA
	ATACGATGATTTTAAGTCATGCTGA
	CGGCAGTATAGCTGAGCAAGTGATT
	CTGCACCGCAGAGGCTACTCAAGTG
	CGTATTGATGTACTTGATGTCGGGC
	AACCAAACTTGGCGTATTGATGTAC
*Staphylococcus aureus*	GTATGATACGACAAAACCACAACGT
	GTATGATACGACAGAACCACAACGT
	CCAGCTAAAGGACAACAAGGTAGCA
	ACCAGCTAAAGGACAACAAGGTAGC
	AAGGACAACAAGGTAGCAAAGGTAG
	AACCAGCTAAAGGACAACAAGGTAG
	TAAAGGACAACAAGGTAGCAAAGGT
	AAAGGACAACAAGGTAGCAAAGGTA
	CAACAAGGTAGCAAAGGTAGTAAGT
	CAACAAGGTAGCAAAGGTAGTGAGT
	AACAACCAGCTAAAGGACAACAAGG
	TGATACGACAAAACCACAACGTATG
	TGATACGACAGAACCACAACGTATG
	AACAAGGTAGCAAAGGTAGTAAGTC
	AACAAGGTAGCAAAGGTAGTGAGTC
	AACCGTCTGGCAAACGAATTAATGC
	AACCATCTGGCAAACGAATTAACGC
	AACCATCTGGCAAACGAATTAATGC
	AACCGTCTGGCAAACGAATTAACGC
	GACTTCACAGCATCAGGTGGCGACG
	TGAATAAACCATCTGGCAAACGAAT
	TGAATAAACCGTCTGGCAAACGAAT
	TGACTTCACAGCATCAGGTGGCGAC
	TCACAGCATCAGGTGGCGACGGATA
*Salmonella* Typhimurium	AGGTGTTTTTACTCACAATCTCGCC
	AAGGTGTTTTTACTCACAATCTCGC
	CGGACGATTAAACCGATAGCCCTGT
	CCAGAACGGCATATTCTTTTGGCGG
	CTTCGAGCAGGATGACCAGAACGGC
	TCTGCTTTGTGTCCCAGCGAAGTCC
	TTCGAGCAGGATGACCAGAACGGCA
	CAGAACGGCATATTCTTTTGGCGGA
	AGAACGGCATATTCTTTTGGCGGAA
	ATACAGCGGGTAAGAGATTCTTCGT
	GAACGGCATATTCTTTTGGCGGAAT
	TGCTTTGTGTCCCAGCGAAGTCCGG
	TCGAGCAGGATGACCAGAACGGCAT
	CGGCGGCTTCGAGCAGGATGACCAG
	CGACATGTTAACGCATTGAGTCAGC
	TCGCTAATCTGCTTTGTGTCCCAGC
	CTCGCTAATCTGCTTTGTGTCCCAG
	TAAACCGATAGCCCTGTCCGTACAG
	AATCTGCTTTGTGTCCCAGCGAAGT
	GCTAATCTGCTTTGTGTCCCAGCGA

### Verification of Specific Probes Among Pathogenic Bacteria

**FIGURE 2:**
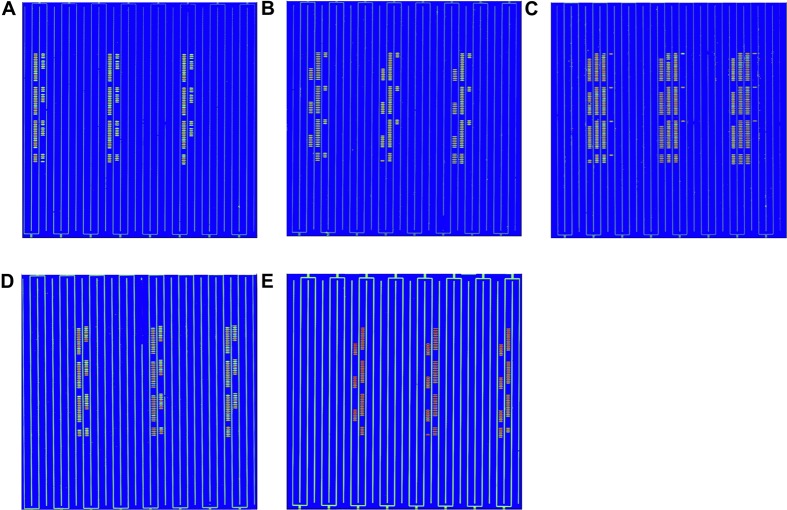
Specific detection by *in situ*-synthesized gene chips of five types of foodborne pathogens. **(A)** *Escherichia coli* O157:H7, **(B)** *Listeria monocytogenes*, **(C)** *Vibrio parahemolyticus*, **(D)** *Staphylococcus aureus*, **(E)** *Salmonella* Typhimurium. The array layout is presented as a list in Supplementary Table S3.

The hybridization specificity was evaluated between the screening probes and the target and non-target pathogen (Figure 2). The hybridization results for each pathogen including EC O157:H7, LM, VP, SA, and ST are shown in Figures 2A–E, respectively. Strong signal values were present on sites between the specific screening probes and the target pathogen. No hybridization signals were found between the screening probes and the non-target pathogens. To determine the stability of the *in situ*-synthesized gene chip for detecting the pathogenic bacteria, the corresponding specific probes were repeatedly synthesized ten times. The signal values remained consistent with each repeated experiment. It demonstrated that the *in situ*-synthesized gene chip including screening probes has a high specificity for detecting EC O157:H7, LM, VP, SA and ST.

### Identification of FoodBorne Pathogens on Fresh-Cut Cantaloupe and Lettuce

**FIGURE 3:**
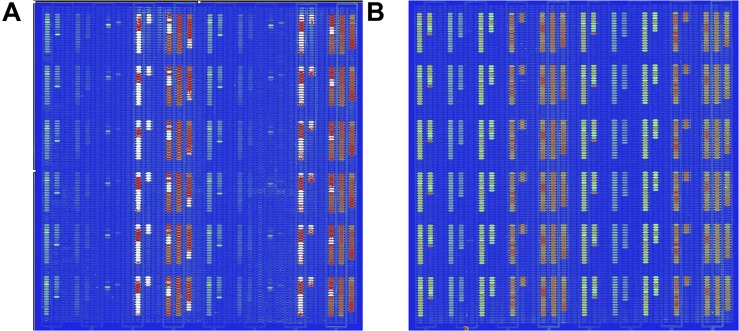
Detection by *in situ*-synthesized gene chips of five types of foodborne pathogens on fresh-cut fruits and vegetables. **(A)** Fresh-cut cantaloupe. **(B)** Fresh-cut lettuce. The array layout is presented as a list in Supplementary Table S4.

The detection of pathogenic bacteria inoculated into fresh-cut cantaloupe and fresh-cut lettuce using the *in situ*-synthesized gene chip is shown in Figure 3. The numbers of EC O157:H7, LM, SA, ST, and VP on fresh-cut cantaloupe were 3.05, 2.19, 2.02, 3.12, and 3.35 log cfu/g, respectively, as determined by colony counting, and strong signal values were found on the regions with probes specific to EC O157:H7, ST, and VP (Figure 3A). Contrarily, low signal values were present on the regions containing probes specific to LM and SA. This discrepancy might be due to cell lysis efficiency during the DNA extraction for the Gram-positive strains (LM and SA). The low abundance of LM and SA might also be the main factor for the low signal values on the fresh-cut cantaloupe. Therefore, the sensitivity of the *in situ*-synthesized gene chip for detecting foodborne pathogens inoculated into fresh-cut cantaloupe was ∼ 3 log cfu/g.

The numbers of EC O157:H7, LM, SA, ST, and VP on fresh-cut lettuce were 3.75, 2.77, 3.34, 3.27, and 3.35 log cfu/g, respectively, as measured according to colony counting. Strong, regular, and obvious signals were observed at specific probes from all five pathogenic bacteria on fresh-cut lettuce (Figure 3B). The hybridization signal from 141 specific probes was found on the *in situ*-synthesized gene chips. Especially the signal values of the probe site on the *in situ*-synthesized gene chip detecting LM and SA on fresh-cut lettuce were higher than those on fresh-cut cantaloupe. It might be due to the fact that the population of LM and SA on fresh-cut lettuce was higher than that on fresh-cut cantaloupe. The 141 specific probes were repeatedly synthesized 12 times on the *in situ*-synthesized gene chips. The signal value remained consistent with each repeated experiment, demonstrating that the probes designed and screened in this study can effectively improve the detection efficiency of these foodborne pathogens on fresh-cut cantaloupe and lettuce.

## Discussion

The foodborne pathogens, primarily LM, ST, SA, VP, and EC O157:H7, are major causes of foodborne diseases worldwide. These pathogens, in particular, are the main reasons responsible for enteritidis, meningitis, and even death (Scallan et al., 2011). The rapid and accurate detection of pathogens on food products and in environmental samples are important for the prevention of outbreaks of foodborne diseases and of spreading of foodborne pathogens (Law et al., 2015). The traditional culture methods have several drawbacks mainly related to the time and labor required, which delay the feedback of information about the pathogens from suspected food (Andrews and Ryan, 2015). The detection techniques for foodborne pathogens have evolved significantly to overcome the limitations of conventional detection in the recent years. Generally, new strategies and methods based on nucleic acid amplification, the recognition of antigen and antibody in immunology, and signal identification with biosensor have been developed in the recent years (Zhao et al., 2014). The DNA microarrays are a comprehensive platform that combine nucleic-acid- and biosensor-based approaches and are a powerful tool for the detection and identification of pathogens on food matrices. The DNA microarrays are useful in the survey of outbreak of foodborne diseases, especially in the screening of foodborne pathogen in a large number of samples (Herrera-Rodriguez et al., 2013). The DNA microarray technology can be used to detect all potential pathogenic bacteria in a sample in a single assay. The microarrays can be designed to detect a broad spectrum of foodborne pathogens without compromising the sensitivity or increasing the time required for detection (Yu et al., 2016). For example, the DNA microarray was found to be more precise compared with the conventional culture for detecting *Campylobacter jejuni* and *Campylobacter coli* (Keramas et al., 2004). Similarly, a mixed genomic microarray was designed and could discriminate between the closely related *L. monocytogenes* isolates within a serotype obtained from similar geographic and epidemiologic sources (Borucki et al., 2003). Another microarray designed for the multi-pathogen identification of 18 pathogenic bacteria was found to be highly specific and sensitive. This array, containing 53,660 probes, could discriminate the amplification products and the false-positive amplification products for positive identifications (Wilson et al., 2002).

However, the microarray technology developed to date faces several challenges for the detection of pathogens as part of food surveillance. The current microarray methods generally require some cultivation of bacteria, DNA preparation, and hybridization during the detection process (Kim et al., 2012; Yu et al., 2016). The detection efficiency may be affected by low amounts of pathogenic bacteria, false-positive results, and inadequate sensitivity (Tarca et al., 2006). It is possible to overcome these challenges and achieve reasonable detection levels by optimizing the sample preparation methodology and by appropriate screening of hybridization probes.

Compared to other nucleic-acid-based platforms, the *in situ*-synthesized gene chips provide greater flexibility in design. The efficacy of this methodology is independent of the efficiency of the target gene amplification, the oligonucleotide length can be easily controlled, and the hybridization probe sequence can be screened from the different virulence gene regions for specific hybridization. Designing and screening specific probes is key to the establishment of *in situ*-synthesis chip technology. However, probe selection is often complicated by the complexity of the target genomes and the differences in sequence composition, which need to be accounted for during probe design. A large number of repeated experiments were performed to verify and screen thousands of effective probes (Gard et al., 2009). High-density *in situ*-synthesis microarrays were used to screen the whole genome of the target pathogens and yielded effective probe hybridization. The tiling probe design was used to screen and verify a single experiment, which saves time and enhances the efficiency of the *in situ*-synthesized microarrays in this study. The high resolution of the tiling arrays was also exploited in this study to differentiate between foodborne pathogens, which is particularly challenging for other traditional methods. The method of tiling probe array on the gene chip expanded the resequencing technology, and the main characteristic was the higher efficiency to identify each base of the oligonucleotide sequence for the detection of base mutation sites (Chee et al., 1996; Winzeler et al., 1998; Jackson et al., 2007). Although the approach of tiling probe array has been developed for decades, there is surprisingly little information about the application of this approach to food safety. In the current study, the probes were arranged as a tiling array on an *in situ*-synthesis microarray platform. The tiling array platform consisted of approximately 3,227 oligonucleotides with 1-bp intervals between the probes. This interval provided redundancy that allowed the detection of specific genes from the foodborne pathogens and also exposed the sites of possible genetic changes that may have altered the genomic sequence of the pathogens. The approach of tiling probe array has the advantage for discovering specific probes to identify virulence genes from the target bacteria. In the tiling array, a specific gene sequence in the genome is identified by comparing the hybridization signal of a target foodborne pathogen to that of a reference strain by matching the probe sequences, and 141 specific probes were obtained from the five foodborne pathogens in this study. This method enabled the detection of multiple target pathogens using customized tiling arrays in contrast to most pathogen detection methods that focus on the detection of pathogens individually. Other research developed a DNA microarray containing the random genomic DNA fragments of *L. monocytogenes* or four virulence genes of *E. coli* O157:H7, which can only detect *L. monocytogenes* or *E. coli* O157:H7 and distinguish it from other foodborne pathogens (Bang et al., 2013; Call et al., 2001). It is obvious that the *in situ*-synthesized gene chip with a tiling probe array contains a more specific probe which hybridizes with the multiplex PCR amplicon of the foodborne pathogen in this study. It can enhance the accuracy and the efficacy of detecting the target foodborne pathogens.

In our arrays, the species of a foodborne pathogen could be identified by the presence of virulence genes related to pathogenicity. The primers and probes designed to target the bacterial virulence genes were able to enhance the specificity for detection. The virulence genes screened in this research have shown a strong specificity toward LM, ST, SA, VP, and EC O157:H7. The gene targets were obtained by multiple comparisons and were based on the whole genome sequences of the five target foodborne pathogens. The five target genes, *hlyA* from LM, *invA* from ST, sash from SA, *comEC/rec2* from VP, and *wzy* from EC O157:H7, were selected for the design of the primers and probes. The amplification results of this detection system had a strong specificity compared with those of other studies based on random amplification (Poretsky et al., 2014). At the species level, the 16S rRNA gene, as the target gene, has been used to detect the pathogens, however, the detection accuracy of 16s rRNA was limited for discriminating at or below the species level due to the low base substitution mutation rates (Ruan et al., 2017; Liu et al., 2019). The *in situ*-synthesis microarray detection platform for the foodborne pathogens is based on nucleic acid hybridization technology rather than on a single marker gene. Multiplex PCR was used to prepare the amplification products for probe hybridization. The reliability and the accuracy of the combined microarray chip and multiplex PCR detection system were further enhanced by the simultaneous identification of the specific regions of the multiple pathogens (Wilson et al., 2002). However, there may be variations in the genes of the different pathogenic bacteria, resulting in mismatches between some probes and the products of PCR. Therefore, the multiple probes were screened for each strain to ensure a specific identification of the target strain. Ultimately, one signal between the probe and the PCR product could be utilized to confirm the presence of the target strain.

In addition, the ingredient in fruits and vegetables also negatively impact the detection efficiency of the *in situ*-synthesized gene chip. The interference from inhibitors, such as humic acid, has been noted as a critical issue by researchers in identifying pathogens in the samples using other detection platforms, such as PCR (Loge et al., 2002). Such inhibitors can often be carried over during DNA extraction, which is the first step in many analytical protocols. However, the purity and quality of DNA is an important factor for DNA microarray analysis (Vital et al., 2016). In fact, the interference from element in food matrices to DNA has not been avoided during some DNA extraction methods, such as column-based extraction, magnetic bead-based extraction, and even automated extraction. In our detection platform, the filtration system effectively removed the micro-particles and the flocculent precipitates after the homogenization of fruits and vegetables, thus reducing the interference of fruit and vegetable DNA to subsequent microbial detection. Our extraction protocol can be used as a sort of a bacterial enrichment device to maximize the enrichment of pathogenic foodborne microorganisms in fruits and vegetables and improve the efficiency and sensitivity of positive detection. This effectively shortens the culture time required for microbial growth.

The application of DNA microarray technology for detecting foodborne pathogen on food matrix still has some difficulties to be resolved. The false-positive result is the major problem while identifying the multiple foodborne pathogens by DNA hybridization. In the current study, the combination of tiling probe array and *in situ*-synthesized gene chip enhanced the accuracy and efficiency. There are also other methods, like digital PCR technology, nanotechnology platform, and biosensor chip technology, that may contribute to further improvements in the detection of DNA from food samples combined with microarrays in the future (Avarre et al., 2007). The *in situ*-synthesized gene chip method provided a comprehensive, highly efficient, and rapid detection for foodborne pathogen. In the case of food poison outbreak associated with foodborne pathogen, the detection of *in situ*-synthesized gene chip enables to provide comprehensive data and strategy, thereby performing a suitable treatment for patients and encouraging a rapid recall of the contaminated food.

## Conclusion

This study developed an *in situ*-synthesized gene chip for detecting foodborne pathogens on fresh-cut fruits and vegetables. This assay showed a strong amplification signal and high accuracy. We obtained 141 specific probes by screening 3,227 potential hybridization probes. The detection limit was approximately 3 log cfu/g without culturing, and the detection time was 24 h. The foodborne pathogen detection technology established in this study can effectively detect and monitor the foodborne pathogens on fresh-cut cantaloupe and lettuce during processing, cleaning, fresh-cutting, packaging, storage, logistics, and sale, thereby improving the quality and safety controls of fresh-cut fruits and vegetables.

## Data Availability Statement

Publicly available datasets were analyzed in this study. This data can be found here: The accession number have been included in the Supplementary Table S1.

## Author Contributions

S, KF, and AJ performed the development of molecular biology method, microarray detection, and sequence screening and drafted the manuscript. WH and ZX conceived and designed the experiments and helped to polish the language. YLa, YLi, and YLo participated in sequence alignment and microbial experiments.

## Conflict of Interest

The authors declare that the research was conducted in the absence of any commercial or financial relationships that could be construed as a potential conflict of interest.
