# Supplementary material for: An in situ-Synthesized Gene Chip for the Detection of Food-Borne Pathogens on Fresh-Cut Cantaloupe and Lettuce
Source: Front Microbiol. 2020 Feb 5;10:3089. doi: 10.3389/fmicb.2019.03089 (PMC7012807; doi:10.3389/fmicb.2019.03089)
Supplement: Supplementary file 1 [file Table_1.pdf]

# *Supplementary Material*

**Supplementary Table 1.** The virulence gene sequence of *Listeria monocytogenes*, *Salmonella* Typhimurium, *Staphylococcus aureus*, *Escherichia coli* O157:H7, and *Vibrio parahaemolyticus*

| Strains | Accession on NCBI |
|---|---|
| *Listeria monocytogenes* | NC_017728; NC_013768; NC_017544; NC_018584; NC_012488; NC_003210; NC_022568; NC_017547; NC_017546; NC_011660; NC_021830; NC_017545; NC_021829; NC_018642; NC_020557; NC_017537; NC_020558; NC_018587; NC_018889; NC_017529; NC_002973; NC_019556; NC_018591; NC_018888; NC_018590; NC_018585; NC_018589; NC_018586; NC_018592; NC_018593; NC_013766; |
| *Salmonella* Typhimurium | NC_015761; NC_021870; NC_010067; NC_021820; NC_022991; NC_011148; NC_011149; NC_021817; NC_021844; NC_022241; NC_022248; NC_006855; NC_006856; NC_006905; NC_021818; NC_021819; NC_021845; NC_011294; NC_011274; NC_022221; NC_016831; NC_021810; NC_021811; NC_021841; NC_021869; NC_017623; NC_017624; NC_021812; NC_021813; NC_021842; NC_011081; NC_011082; NC_011083; NC_020306; NC_020307; NC_020308; NC_009140; NC_011079; NC_011080; NC_021902; NC_011147; NC_006511; NC_010102; NC_012124; NC_012125; NC_021984; NC_011092; NC_011093; NC_011094; NC_022525; NC_003198; NC_003384; NC_003385; NC_016825; NC_016832; NC_004631; NC_021176; NC_017046; NC_017054; NC_016855; NC_016856; NC_016854; NC_022544; NC_003197; NC_003277; NC_016810; NC_017718; NC_017719; NC_017720; NC_016857; NC_016858; NC_016859; NC_017675; NC_016860; NC_016861; NC_016862; NC_021151; NC_021155; NC_021156; NC_021157; NC_016863; NC_016864; NC_021814; NC_021815; NC_021816; NC_021843; NT_187069; NT_187070; NT_187071; NT_187072; NT_187073; NT_187074; NT_187075; NT_187076; NT_187077; NT_187078; NT_187079; NT_187080; NT_187081; NT_187082; NT_187083; NT_187084; NT_187085; NT_187086; NT_187087; NT_187088; NT_187089; NT_187090; NT_187091; NT_187092; NT_187093; NT_187094; NT_187095; NT_187096; NT_187097; NT_187098; NT_187099; NT_187100; NT_187101; NT_187102; NT_187103; NT_187104; NT_187105; NT_187106; NT_187107; NT_187108; NT_187109; NT_187110; NT_187111; NT_187112; |

| | |
|---|---|
| | NT_187113; NT_187114; NT_187115; NT_187116; NT_187117; NT_187118; NT_187119; NT_187120; NT_187121; NT_187122; NT_187123; NT_187124; NT_187125; NT_187126; NT_187127; NT_187128; NT_187129; NT_187130; NT_187131; ;NT_187132; NT_187133; NT_187134; NT_187135; |
| *Staphylococcus aureus* | NC_017340; NC_01860; NC_022113; NC_022126; NC_022222; NC_017350; NC_017351; NC_017673; NC_021657; NC_021670; NC_021552; NC_021554; NC_022226; NC_022227; NC_022228; NC_002951; NC_006629; NC_017343; NC_017344; NC_017346; NC_013450; NC_013451; NC_013452; NC_013453; NC_017337; NC_017763; NC_009619; NC_009632; NC_009477; NC_009487; NC_017341; NC_017338; NC_017339; NC_017348; NC_017349; NC_021059; NC_021060; NC_016928; NC_002952; NC_016941; NC_016942; NC_002953; NC_005951; NC_009782; NC_002758; NC_002774; NC_003923; NC_002745; NC_003140; NC_007795; NC_009641; NC_007622; NC_022443; NC_022442; NC_020529; NC_020530; NC_020531; NC_020564; NC_020532; NC_020565; NC_020533; NC_020534; NC_020537; NC_020538; NC_020539; NC_020568; NC_017333; NC_017334; NC_017335; NC_017336; NC_017347; NC_017342; NC_017345; NC_017331; NC_017332; NC_017352; NC_020535; NC_020566; NC_020536; NC_020567; NC_007790; NC_007791; NC_007792; NC_007793; NC_010063; NC_010079; NC_012417; NC_016912; NC_022604; NC_022605; NC_022610; NC_021824; NC_021825; |
| *Vibrio parahaemolyticus* | NC_019955; NC_019971; NC_021821; NC_021822; NC_021823; NC_021826; NC_021827; NC_021828; NC_021834; NC_021837; NC_021838; NC_021839; NC_021840; NC_021847; NC_021848; NC_022045; NC_022046; NC_004603; NC_004605; |
| *Escherichia coli* O157:H7 | NC_011350; NC_011351; NC_011353; NC_002655; NC_007414; NC_013008; NC_013010; NC_002127; |
