# Supplementary material for: An in situ-Synthesized Gene Chip for the Detection of Food-Borne Pathogens on Fresh-Cut Cantaloupe and Lettuce
Source: Front Microbiol. 2020 Feb 5;10:3089. doi: 10.3389/fmicb.2019.03089 (PMC7012807; doi:10.3389/fmicb.2019.03089)
Supplement: Supplementary file 5 [file Table_5.pdf]

# *Supplementary Material*

## Supplementary Table 5. The signal value of top 100 hybridization probe for *Listeria monocytogenes*

| No. | probe Sequence (5' to 3') | Row | Column | Density (mean) | Density (st.dev.) |
|---|---|---|---|---|---|
| 1 | AAACTTCGGCGCAATCAGTGAAGGG | 1 | 112 | 54157.27 | 408.82 |
| 2 | TAATAGCTTGAATGTAAACTTCGGC | 1 | 97 | 49878.87 | 813.49 |
| 3 | GCGCAATCAGTGAAGGGAAAATGCA | 1 | 120 | 48763.42 | 778.08 |
| 4 | TAAACTTCGGCGCAATCAGTGAAGG | 1 | 111 | 47450.44 | 736.57 |
| 5 | GAATGTAAACTTCGGCGCAATCAGT | 1 | 106 | 43614.96 | 1042.45 |
| 6 | TGTAAACTTCGGCGCAATCAGTGAA | 1 | 109 | 43126.29 | 934.23 |
| 7 | AATTGATTATGATGACGAAATGGCT | 1 | 12 | 42322.44 | 1953.43 |
| 8 | GGCGCAATCAGTGAAGGGAAAATGC | 1 | 119 | 41881.64 | 1051.90 |
| 9 | AAATTGATTATGATGACGAAATGGC | 1 | 11 | 41172.20 | 788.66 |
| 10 | TCGGCGCAATCAGTGAAGGGAAAAT | 1 | 117 | 40730.67 | 756.92 |
| 11 | GATTATGATGACGAAATGGCTTACA | 1 | 16 | 39701.06 | 660.53 |
| 12 | TGATTATGATGACGAAATGGCTTAC | 1 | 15 | 39387.86 | 473.13 |
| 13 | AGCTTGAATGTAAACTTCGGCGCAA | 1 | 101 | 39176.09 | 698.85 |
| 14 | TGATGACGAAATGGCTTACAGTGAA | 1 | 21 | 38956.58 | 780.08 |
| 15 | ATGACGAAATGGCTTACAGTGAATC | 1 | 23 | 38706.56 | 621.10 |

| | | | | | |
|---|---|---|---|---|---|
| 16 | ATGATGACGAAATGGCTTACAGTGA | 1 | 20 | 38459.51 | 339.06 |
| 17 | GATGACGAAATGGCTTACAGTGAAT | 1 | 22 | 38056.66 | 425.35 |
| 18 | CTTGAATGTAAACTTCGGCGCAATC | 1 | 103 | 37725.03 | 552.94 |
| 19 | TTATGATGACGAAATGGCTTACAGT | 1 | 18 | 37695.12 | 599.67 |
| 20 | TTCGGCGCAATCAGTGAAGGGAAAA | 1 | 116 | 36663.90 | 2738.72 |
| 21 | TTGAATGTAAACTTCGGCGCAATCA | 1 | 104 | 35695.72 | 773.28 |
| 22 | GACGAAATGGCTTACAGTGAATCAC | 1 | 25 | 34934.98 | 915.98 |
| 23 | CGCAATCAGTGAAGGGAAAATGCAA | 1 | 121 | 34709.13 | 795.65 |
| 24 | CGAAATGGCTTACAGTGAATCACAA | 1 | 27 | 34642.97 | 1387.81 |
| 25 | GACCTTCCAGATTTTTCGGCAAAGC | 2 | 81 | 48961.96 | 593.62 |
| 26 | TTAATGAACCTACAAGACCTTCCAG | 2 | 66 | 43824.46 | 286.20 |
| 27 | CAAGACCTTCCAGATTTTTCGGCAA | 2 | 78 | 42094.60 | 840.94 |
| 28 | ATGTTAATGAACCTACAAGACCTTC | 2 | 63 | 40879.47 | 964.64 |
| 29 | TAATGAACCTACAAGACCTTCCAGA | 2 | 67 | 40823.62 | 559.47 |
| 30 | AAAGCTGTTACTAAAGAGCAGTTGC | 2 | 101 | 40750.16 | 494.54 |
| 31 | TCGGCAAAGCTGTTACTAAAGAGCA | 2 | 96 | 38700.01 | 429.23 |
| 32 | TTCCAGATTTTTCGGCAAAGCTGTT | 2 | 85 | 38680.92 | 413.38 |
| 33 | TACAAGACCTTCCAGATTTTTCGGC | 2 | 76 | 37798.92 | 906.89 |
| 34 | GTGAAGGGAAAATGCAAGAAGAAGT | 2 | 6 | 37452.80 | 720.20 |
| 35 | CAAAGCTGTTACTAAAGAGCAGTTG | 2 | 100 | 37275.36 | 477.93 |
| 36 | CCTTCCAGATTTTTCGGCAAAGCTG | 2 | 83 | 37274.01 | 641.82 |

| | | | | | |
|---|---|---|---|---|---|
| 37 | ATCAGTGAAGGGAAAATGCAAGAAG | 2 | 2 | 36047.72 | 559.05 |
| 38 | AATGTTAATGAACCTACAAGACCTT | 2 | 62 | 36003.09 | 993.74 |
| 39 | TGAATGTTAATGAACCTACAAGACC | 2 | 60 | 35776.23 | 1036.60 |
| 40 | TCAGTGAAGGGAAAATGCAAGAAGA | 2 | 3 | 35411.77 | 295.72 |
| 41 | GGGAAAATGCAAGAAGAAGTCATTA | 2 | 11 | 35142.14 | 984.31 |
| 42 | TGAAGGGAAAATGCAAGAAGAAGTC | 2 | 7 | 34834.96 | 645.80 |
| 43 | AGCTGTTACTAAAGAGCAGTTGCAA | 2 | 103 | 34760.36 | 602.73 |
| 44 | GAAGGGAAAATGCAAGAAGAAGTCA | 2 | 8 | 34492.52 | 1146.82 |
| 45 | AAGATGAAGTTCAAATCATCGACGG | 4 | 89 | 33861.45 | 858.63 |
| 46 | GCTGTTATTAAAAACAACTCAGAAT | 5 | 120 | 36285.97 | 347.36 |
| 47 | TTAGCTGTTATTAAAAACAACTCAG | 5 | 117 | 34952.95 | 573.86 |
| 48 | AGCTGTTATTAAAAACAACTCAGAA | 5 | 119 | 34369.63 | 400.88 |
| 49 | TCGATCACTCTGGAGGATACGTTGC | 6 | 62 | 43012.61 | 699.87 |
| 50 | CTCTGGAGGATACGTTGCTCAATTC | 6 | 69 | 41139.30 | 1021.09 |
| 51 | CACTCTGGAGGATACGTTGCTCAAT | 6 | 67 | 40332.06 | 635.93 |
| 52 | CTGGAGGATACGTTGCTCAATTCAA | 6 | 71 | 39675.04 | 466.29 |
| 53 | CATCGATCACTCTGGAGGATACGTT | 6 | 60 | 38757.87 | 587.24 |
| 54 | AACATCGATCACTCTGGAGGATACG | 6 | 58 | 38166.81 | 630.08 |
| 55 | GATCACTCTGGAGGATACGTTGCTC | 6 | 64 | 38078.05 | 990.56 |
| 56 | ACTCTGGAGGATACGTTGCTCAATT | 6 | 68 | 37985.46 | 1225.57 |
| 57 | ACATCGATCACTCTGGAGGATACGT | 6 | 59 | 37774.19 | 713.79 |

| | | | | | |
|---|---|---|---|---|---|
| 58 | TGATCCTGAAGGTAACGAAATTGTT | 6 | 120 | 37149.70 | 1012.94 |
| 59 | TCTGGAGGATACGTTGCTCAATTCA | 6 | 70 | 36956.08 | 733.30 |
| 60 | TGGAGGATACGTTGCTCAATTCAAC | 6 | 72 | 36783.69 | 388.24 |
| 61 | ATCGATCACTCTGGAGGATACGTTG | 6 | 61 | 34981.16 | 705.68 |
| 62 | CAACTCAGAATATATTGAAACAACT | 6 | 6 | 34180.21 | 1068.91 |
| 63 | CTCAGAATATATTGAAACAACTTCA | 6 | 9 | 34057.54 | 496.94 |
| 64 | CCTGAAGGTAACGAAATTGTTCAAC | 6 | 124 | 33860.72 | 874.08 |
| 65 | TATGATCCTGAAGGTAACGAAATTG | 6 | 118 | 33830.15 | 513.89 |
| 66 | GTCCATCTATTTGCCAGGTAACGCG | 7 | 70 | 52325.01 | 1093.49 |
| 67 | ATCGTCCATCTATTTGCCAGGTAAC | 7 | 67 | 47427.92 | 1056.16 |
| 68 | CATCGTCCATCTATTTGCCAGGTAA | 7 | 66 | 47053.83 | 988.68 |
| 69 | TGCACTGGTTTAGCTTGGGAATGGT | 7 | 122 | 46964.19 | 925.72 |
| 70 | GCTAGCTCATTTCACATCGTCCATC | 7 | 52 | 46062.84 | 272.58 |
| 71 | GAATGCACTGGTTTAGCTTGGGAAT | 7 | 119 | 45670.75 | 625.64 |
| 72 | TTTCACATCGTCCATCTATTTGCCA | 7 | 61 | 45473.84 | 588.33 |
| 73 | GCACTGGTTTAGCTTGGGAATGGTG | 7 | 123 | 44316.06 | 892.22 |
| 74 | CACATCGTCCATCTATTTGCCAGGT | 7 | 64 | 44121.28 | 1217.07 |
| 75 | GTTTACGCTAAAGAATGCACTGGTT | 7 | 107 | 44082.12 | 488.12 |
| 76 | TACGCTAAAGAATGCACTGGTTTAG | 7 | 110 | 43746.56 | 377.15 |
| 77 | ACATCGTCCATCTATTTGCCAGGTA | 7 | 65 | 42337.86 | 1459.58 |
| 78 | CCATCTATTTGCCAGGTAACGCGAG | 7 | 72 | 41663.89 | 678.48 |

| | | | | | |
|---|---|---|---|---|---|
| 79 | AGCTAGCTCATTTCACATCGTCCAT | 7 | 51 | 41296.40 | 245.06 |
| 80 | AAGCTAGCTCATTTCACATCGTCCA | 7 | 50 | 41097.95 | 360.81 |
| 81 | TGTTCAACATAAAAACTGGAGCGAA | 7 | 13 | 41091.04 | 1283.57 |
| 82 | ATGCACTGGTTTAGCTTGGGAATGG | 7 | 121 | 41087.04 | 1083.78 |
| 83 | CGCTAAAGAATGCACTGGTTTAGCT | 7 | 112 | 40514.53 | 433.00 |
| 84 | TCAACATAAAAACTGGAGCGAAAAC | 7 | 16 | 40380.97 | 855.59 |
| 85 | ACGCTAAAGAATGCACTGGTTTAGC | 7 | 111 | 39953.58 | 531.44 |
| 86 | GTTCAACATAAAAACTGGAGCGAAA | 7 | 14 | 39675.72 | 1232.75 |
| 87 | TTGTTCAACATAAAAACTGGAGCGA | 7 | 12 | 39626.36 | 1352.69 |
| 88 | TCACATCGTCCATCTATTTGCCAGG | 7 | 63 | 38831.47 | 780.73 |
| 89 | CAACATAAAAACTGGAGCGAAAACA | 7 | 17 | 38533.93 | 578.65 |
| 90 | TTACGCTAAAGAATGCACTGGTTTA | 7 | 109 | 38444.99 | 544.63 |
| 91 | ACATAAAAACTGGAGCGAAAACAAT | 7 | 19 | 38259.83 | 495.84 |
| 92 | GCTCATTTCACATCGTCCATCTATT | 7 | 56 | 37763.10 | 605.90 |
| 93 | TAAAGAATGCACTGGTTTAGCTTGG | 7 | 115 | 37115.97 | 703.01 |
| 94 | CATAAAAACTGGAGCGAAAACAATA | 7 | 20 | 36430.89 | 299.37 |
| 95 | TTCAACATAAAAACTGGAGCGAAAA | 7 | 15 | 36311.92 | 1093.27 |
| 96 | CTAGCTCATTTCACATCGTCCATCT | 7 | 53 | 36077.61 | 579.51 |
| 97 | TAATGTTTACGCTAAAGAATGCACT | 7 | 103 | 35805.73 | 488.76 |
| 98 | AACATAAAAACTGGAGCGAAAACAA | 7 | 18 | 35640.69 | 506.38 |
| 99 | GAAGCTAGCTCATTTCACATCGTCC | 7 | 49 | 35465.19 | 433.67 |

| | | | | | |
|---|---|---|---|---|---|
| 100 | CTAAAGAATGCACTGGTTTAGCTTG | 7 | 114 | 34919.22 | 699.92 |
