# Supplementary material for: An in situ-Synthesized Gene Chip for the Detection of Food-Borne Pathogens on Fresh-Cut Cantaloupe and Lettuce
Source: Front Microbiol. 2020 Feb 5;10:3089. doi: 10.3389/fmicb.2019.03089 (PMC7012807; doi:10.3389/fmicb.2019.03089)
Supplement: Supplementary file 6 [file Table_6.pdf]

Supplementary Material

Supplementary Table 6. The signal value of top 100 hybridization probe for *Salmonella* Typhimurium

No.	Probe Sequence (5' to 3')	Row	Column	Density (mean)	Density (st.dev.)
1	GGTGTTTTTACTCACAATCTCGCCG	110	14	37834.88	632.78
2	GGACGATTAAACCGATAGCCCTGTC	92	13	35045.35	593.82
3	AGGTGTTTTTACTCACAATCTCGCC	109	14	35042.88	506.70
4	CCGGACGATTAAACCGATAGCCCTG	90	13	34730.03	134.89
5	AAGGTGTTTTTACTCACAATCTCGC	108	14	34469.45	527.99
6	CGGACGATTAAACCGATAGCCCTGT	91	13	34137.31	178.06
7	ATTAAACCGATAGCCCTGTCCGTAC	97	13	33097.17	333.80
8	CGACCGGACGATTAAACCGATAGCC	87	13	32931.44	363.18
9	ACCGGACGATTAAACCGATAGCCCT	89	13	32835.74	160.21
10	CCAGAACGGCATATTCTTTTGGCGG	26	15	32536.39	511.58
11	ACCAGAACGGCATATTCTTTTGGCG	25	15	32235.85	483.37
12	GATTAAACCGATAGCCCTGTCCGTA	96	13	32130.38	366.39
13	CGATTAAACCGATAGCCCTGTCCGT	95	13	31969.06	269.60
14	TAAGGTGTTTTTACTCACAATCTCG	107	14	31752.33	376.90
15	GTAAGGTGTTTTTACTCACAATCTC	106	14	31656.25	657.95

16	GTGTTTTTACTCACAATCTCGCCGG	111	14	31596.24	571.34
17	TTAAACCGATAGCCCTGTCCGTACA	98	13	31511.11	307.74
18	TACAGCGGGTAAGAGATTCTTCGTT	52	14	31227.26	420.35
19	CTTCGAGCAGGATGACCAGAACGGC	11	15	31169.31	740.15
20	CTGCTTTGTGTCCCAGCGAAGTCCG	34	10	31001.34	627.57
21	TCTGCTTTGTGTCCCAGCGAAGTCC	33	10	30808.44	770.82
22	TTCGAGCAGGATGACCAGAACGGCA	12	15	30802.47	819.08
23	GATTGAACCTGATCCTGCATCTGAA	5	13	30641.69	677.22
24	CAGAACGGCATATTCTTTTGGCGGA	27	15	30589.15	626.71
25	CATGTTAACGCATTGAGTCAGCAAT	14	9	30461.72	587.09
26	GGTCCAGTAAGGTGTTTTTACTCAC	100	14	30421.90	483.50
27	GACGATTAAACCGATAGCCCTGTCC	93	13	30309.12	225.95
28	AGAACGGCATATTCTTTTGGCGGAA	28	15	30195.06	559.44
29	ATACATCTGAGCAAAAGATTCGCAA	62	10	30169.06	391.20
30	AATACATCTGAGCAAAAGATTCGCA	61	10	29902.79	357.95
31	GCTTTGTGTCCCAGCGAAGTCCGGG	36	10	29879.50	359.06
32	ATCTGCTTTGTGTCCCAGCGAAGTC	32	10	29759.97	458.07
33	ATACAGCGGGTAAGAGATTCTTCGT	51	14	29732.68	590.77
34	GGATTGAACCTGATCCTGCATCTGA	4	13	29691.56	400.63
35	GCGAAGTCCGGGAATACATCTGAGC	49	10	29605.25	581.01
36	GAACGGCATATTCTTTTGGCGGAAT	29	15	29564.48	352.74

37	AGTAAGGTGTTTTTACTCACAATCT	105	14	29511.36	371.34
38	TGCTTTGTGTCCCAGCGAAGTCCGG	35	10	29476.02	683.60
39	GGCTTCGAGCAGGATGACCAGAACG	9	15	29449.31	394.27
40	ACGACCGGACGATTAAACCGATAGC	86	13	29311.58	185.99
41	CGGCTTCGAGCAGGATGACCAGAAC	8	15	29261.09	354.77
42	TCGAGCAGGATGACCAGAACGGCAT	13	15	29196.78	794.25
43	ACGATTAAACCGATAGCCCTGTCCG	94	13	29165.76	292.40
44	GCGGCTTCGAGCAGGATGACCAGAA	7	15	29165.09	588.64
45	GGCGGCTTCGAGCAGGATGACCAGA	6	15	29089.92	390.59
46	GCTTCGAGCAGGATGACCAGAACGG	10	15	29034.93	534.81
47	CGGCGGCTTCGAGCAGGATGACCAG	5	15	29017.99	419.01
48	GAATACATCTGAGCAAAAGATTCGC	60	10	29012.53	289.45
49	ATTGAACCTGATCCTGCATCTGAAA	6	13	28921.81	370.22
50	AATACAGCGGGTAAGAGATTCTTCG	50	14	28890.95	682.38
51	CGGATTGAACCTGATCCTGCATCTG	3	13	28839.78	610.63
52	ACATGTTAACGCATTGAGTCAGCAA	13	9	28783.72	617.10
53	GTAATGCAGACTCTCGGATTGAACC	117	12	28770.04	376.86
54	ACAGCGGGTAAGAGATTCTTCGTTA	53	14	28759.50	444.69
55	CGACATGTTAACGCATTGAGTCAGC	11	9	28754.73	342.05
56	ACATCTGAGCAAAAGATTCGCAATT	64	10	28710.97	162.19
57	GACCAGAACGGCATATTCTTTTGGC	24	15	28643.56	786.04

58	GCAGACTCTCGGATTGAACCTGATC	122	12	28635.91	371.28
59	CTCGGATTGAACCTGATCCTGCATC	1	13	28603.01	461.14
60	TCGGATTGAACCTGATCCTGCATCT	2	13	28594.99	336.21
61	TCGCTAATCTGCTTTGTGTCCCAGC	26	10	28512.73	630.08
62	CTCGCTAATCTGCTTTGTGTCCCAG	25	10	28447.94	776.64
63	TAAACCGATAGCCCTGTCCGTACAG	99	13	28343.06	644.15
64	TGTTAACGCATTGAGTCAGCAATTT	16	9	28318.41	621.45
65	GTCCAGTAAGGTGTTTTTACTCACA	101	14	28223.29	296.95
66	AATGCAGACTCTCGGATTGAACCTG	119	12	28215.06	251.52
67	TTGAACCTGATCCTGCATCTGAAAA	7	13	28210.64	239.66
68	AATCTGCTTTGTGTCCCAGCGAAGT	31	10	28194.64	895.55
69	TAATGCAGACTCTCGGATTGAACCT	118	12	28151.56	589.27
70	CCAGCGAAGTCCGGGAATACATCTG	46	10	28147.53	494.10
71	AGTAATGCAGACTCTCGGATTGAAC	116	12	28119.98	238.22
72	GCTAATCTGCTTTGTGTCCCAGCGA	28	10	28119.22	744.05
73	CAGCGGGTAAGAGATTCTTCGTTAA	54	14	28109.53	318.13
74	GCTCAAGAATATCCTTAACACTGCG	17	12	28060.95	522.76
75	TGCAGACTCTCGGATTGAACCTGAT	121	12	28044.52	253.35
76	CAAGAATATCCTTAACACTGCGGCA	20	12	27876.06	812.84
77	GACCGGACGATTAAACCGATAGCCC	88	13	27842.70	191.95
78	GTAACGATGCTCTTTATCTTCCGAC	5	14	27818.55	395.30

79	TGTAACGATGCTCTTTATCTTCCGA	4	14	27808.86	434.48
80	TCAAGAATATCCTTAACACTGCGGC	19	12	27800.94	333.66
81	CGTCGTAAGGCATAAATACAGCGGG	36	14	27799.83	176.57
82	AACGATGCTCTTTATCTTCCGACAG	7	14	27654.05	285.41
83	ATGCAGACTCTCGGATTGAACCTGA	120	12	27604.09	201.30
84	CGAAGTCCGGGAATACATCTGAGCA	50	10	27573.55	454.01
85	CAGTAAGGTGTTTTTACTCACAATC	104	14	27553.82	263.40
86	CGAGCAGGATGACCAGAACGGCATA	14	15	27543.83	568.05
87	ATGTTAACGCATTGAGTCAGCAATT	15	9	27516.81	561.66
88	AAATACAGCGGGTAAGAGATTCTTC	49	14	27488.85	453.56
89	TCTGAGCAAAAGATTCGCAATTTTG	67	10	27464.48	245.51
90	TGGTCCAGTAAGGTGTTTTTACTCA	99	14	27258.43	363.31
91	GACATGTTAACGCATTGAGTCAGCA	12	9	27210.71	586.34
92	CCACTACGACCGGACGATTAAACCG	81	13	27208.61	310.62
93	AAACCGATAGCCCTGTCCGTACAGT	100	13	27206.63	182.08
94	CAGACTCTCGGATTGAACCTGATCC	123	12	27156.88	357.98
95	ATGTAACGATGCTCTTTATCTTCCG	3	14	27060.55	322.87
96	ACTCTCGGATTGAACCTGATCCTGC	126	12	26993.44	401.05
97	TCTTCGTAATGGTCACCGGCAGCAC	47	12	26959.31	544.78
98	TGAACCTGATCCTGCATCTGAAAAG	8	13	26933.25	355.91
99	GTTCTTCGTAATGGTCACCGGCAGC	45	12	26789.31	904.09

100	GATAGAGTAATGCAGACTCTCGGAT	111	12	26771.69	222.81
