# Supplementary material for: An in situ-Synthesized Gene Chip for the Detection of Food-Borne Pathogens on Fresh-Cut Cantaloupe and Lettuce
Source: Front Microbiol. 2020 Feb 5;10:3089. doi: 10.3389/fmicb.2019.03089 (PMC7012807; doi:10.3389/fmicb.2019.03089)
Supplement: Supplementary file 7 [file Table_7.pdf]

Supplementary Material

Supplementary Table 7. The signal value of top 100 hybridization probe for *Staphylococcus aureus*

No.	Probe Sequence (5' to 3')	Row	Column	Density (mean)	Density (st.dev.)
1	AGTATGATACGACAGAACCACAACG	10	21	28365.96	315.21
2	GTATGATACGACAGAACCACAACGT	11	21	27089.46	441.26
3	CCAGCTAAAGGACAACAAGGTAGCA	43	18	25764.96	574.60
4	GGACAACAAGGTAGCAAAGGTAGTG	52	18	25702.19	382.19
5	ACCAGCTAAAGGACAACAAGGTAGC	42	18	25529.87	460.65
6	AAGGACAACAAGGTAGCAAAGGTAG	50	18	25209.65	512.78
7	ACAACCAGCTAAAGGACAACAAGGT	39	18	25029.23	338.58
8	AACCAGCTAAAGGACAACAAGGTAG	41	18	24745.42	484.19
9	TATGATACGACAGAACCACAACGTA	12	21	24563.73	309.54
10	AGGACAACAAGGTAGCAAAGGTAGT	51	18	24506.26	520.68
11	CAACCAGCTAAAGGACAACAAGGTA	40	18	24488.27	420.74
12	GCTAAAGGACAACAAGGTAGCAAAG	46	18	24335.10	357.77
13	TAAAGGACAACAAGGTAGCAAAGGT	48	18	24091.43	466.91
14	CAGCTAAAGGACAACAAGGTAGCAA	44	18	24074.33	467.20
15	AAGTATGATACGACAGAACCACAAC	9	21	24065.12	274.06

16	CTAAAGGACAACAAGGTAGCAAAGG	47	18	23773.67	402.24
17	AAAGGACAACAAGGTAGCAAAGGTA	49	18	23715.75	422.71
18	GAACAACCAGCTAAAGGACAACAAG	37	18	23216.00	494.81
19	AGCTAAAGGACAACAAGGTAGCAAA	45	18	23155.03	286.30
20	CAACAAGGTAGCAAAGGTAGTGAGT	55	18	23125.83	417.84
21	AACAACCAGCTAAAGGACAACAAGG	38	18	22963.92	445.02
22	ATGATACGACAGAACCACAACGTAT	13	21	22751.83	214.84
23	CGATGAATCCAGCGAAACAACCAGC	79	21	22664.41	355.65
24	GATGAATCCAGCGAAACAACCAGCG	80	21	22645.74	432.51
25	GCGATGAATCCAGCGAAACAACCAG	78	21	22633.07	521.17
26	GGACAACAAGGTAGCAAAGGTAGTA	25	21	22605.50	316.23
27	AACCAGCAGTAAGTGAACAACCAGC	23	18	22602.90	554.77
28	TGAACAACCAGCTAAAGGACAACAA	36	18	22594.29	366.42
29	TGATACGACAGAACCACAACGTATG	14	21	22569.51	145.26
30	GACAAAGCGATGAATCCAGCGAAAC	72	21	22545.97	436.16
31	GCTAAGTATGATACGACAGAACCAC	128	20	22426.14	693.52
32	GTGAACAACCAGCTAAAGGACAACA	35	18	22384.80	268.12
33	AGTGAACAACCAGCTAAAGGACAAC	34	18	22236.31	273.55
34	GATCCAGCGAAACAACCAGCGACAG	16	19	22101.69	458.75
35	GACAACAAGGTAGCAAAGGTAGTGA	53	18	21870.07	481.83
36	AAAGCGATGAATCCAGCGAAACAAC	75	21	21862.85	551.35

37	AGCGATGAATCCAGCGAAACAACCA	77	21	21807.30	348.50
38	CGACAAAGCGATGAATCCAGCGAAA	71	21	21749.59	515.68
39	TAAGTATGATACGACAGAACCACAA	8	21	21705.54	322.72
40	CAAAGCGATGAATCCAGCGAAACAA	74	21	21651.19	597.84
41	ACGACAAAGCGATGAATCCAGCGAA	70	21	21647.55	510.65
42	TCCAGCGAAACAACCAGCGACAGGT	18	19	21636.11	601.99
43	ACCAGCAGTAAGTGAACAACCAGCT	24	18	21592.36	205.06
44	AGACAAAGCGATGAATCCAGCGAAA	69	21	21563.35	339.70
45	CTAAGTATGATACGACAGAACCACA	7	21	21557.67	874.39
46	AAGACAAAGCGATGAATCCAGCGAA	68	21	21533.84	305.47
47	TGATGAATCCAGCGAAACAACCAGC	67	21	21515.79	299.28
48	ACAAAGCGATGAATCCAGCGAAACA	73	21	21413.38	662.88
49	ACAACAAGGTAGCAAAGGTAGTGAG	54	18	21255.22	210.31
50	AAACCAGCAGTAAGTGAACAACCAG	22	18	20982.53	390.63
51	AAGCGATGAATCCAGCGAAACAACC	76	21	20870.46	585.42
52	ATCCAGCGAAACAACCAGCGACAGG	17	19	20788.28	589.30
53	GTGATGAATCCAGCGAAACAACCAG	66	21	20521.70	299.03
54	GCGAAACAACCAGCGACAGGTAAAG	22	19	20479.55	542.60
55	ACTTCACAGCATCAGGTGGCGACGG	79	16	20365.43	598.78
56	CCAGCAGTAAGTGAACAACCAGCTA	25	18	20356.88	266.95
57	AACAAGGTAGCAAAGGTAGTGAGTC	56	18	20212.52	288.25

58	AACCGTCTGGCAAACGAATTAACGC	65	20	20156.69	625.19
59	GACAACAAGGTAGCAAAGGTAGTAA	26	21	20134.80	341.38
60	GTAAACCAGCAGTAAGTGAACAACC	20	18	20112.44	694.36
61	GACTTCACAGCATCAGGTGGCGACG	78	16	20109.17	543.69
62	GGATCCAGCGAAACAACCAGCGACA	15	19	20100.76	400.77
63	TGAATAAACCGTCTGGCAAACGAAT	59	20	20082.81	383.77
64	GATACGACAGAACCACAACGTATGT	15	21	20069.47	245.71
65	TAAACCAGCAGTAAGTGAACAACCA	21	18	20022.61	293.14
66	CTTCACAGCATCAGGTGGCGACGGA	80	16	20016.84	526.94
67	TGACTTCACAGCATCAGGTGGCGAC	77	16	19893.87	620.45
68	TTCACAGCATCAGGTGGCGACGGAT	81	16	19794.67	466.51
69	CAAGGTAGCAAAGGTAGTGAGTCTG	58	18	19777.41	231.97
70	CACAGCATCAGGTGGCGACGGATAT	83	16	19674.08	442.49
71	TGGATCCAGCGAAACAACCAGCGAC	14	19	19587.34	470.48
72	AGCTAAGTATGATACGACAGAACCA	127	20	19475.99	597.05
73	GGTAAACCAGCAGTAAGTGAACAAC	19	18	19446.51	441.89
74	TCACAGCATCAGGTGGCGACGGATA	82	16	19384.14	528.22
75	AATGACTTCACAGCATCAGGTGGCG	75	16	19334.77	519.33
76	ACAAGGTAGCAAAGGTAGTGAGTCT	57	18	19319.31	424.91
77	CCAGCGAAACAACCAGCGACAGGTA	19	19	19296.38	545.68
78	ATGACTTCACAGCATCAGGTGGCGA	76	16	19222.69	600.60

79	CAGCATCAGGTGGCGACGGATATAG	85	16	19133.91	501.29
80	GTAAGTGAACAACCAGCTAAAGGAC	31	18	19120.54	240.29
81	CGAAACAACCAGCGACAGGTAAAGT	23	19	19109.90	655.64
82	ACAGCATCAGGTGGCGACGGATATA	84	16	19042.73	441.70
83	AAGTGAACAACCAGCTAAAGGACAA	33	18	18989.33	422.75
84	CCGTCTGGCAAACGAATTAACGCTA	67	20	18980.69	419.74
85	TATGAATAAACCGTCTGGCAAACGA	57	20	18939.08	302.75
86	GAAACAACCAGCGACAGGTAAAGTT	24	19	18928.33	717.67
87	TAGCTAAGTATGATACGACAGAACC	126	20	18890.81	621.17
88	AGTGATGAATCCAGCGAAACAACCA	65	21	18881.69	303.15
89	CAACAAGGTAGCAAAGGTAGTAAGT	28	21	18838.17	388.93
90	GAGACAGGTAAGTTTGAAAATATTG	124	15	18737.13	798.97
91	ACCGTCTGGCAAACGAATTAACGCT	66	20	18704.95	645.20
92	TAAAGAGACAGGTAAGTTTGAAAAT	120	15	18648.17	557.36
93	GAATGACTTCACAGCATCAGGTGGC	74	16	18605.12	513.14
94	ATGGATCCAGCGAAACAACCAGCGA	13	19	18514.14	153.54
95	ACAACCAATTGGTGACGACAAAGCG	120	18	18501.25	187.34
96	ATGAATAAACCGTCTGGCAAACGAA	58	20	18383.62	340.97
97	CAGCGAAACAACCAGCGACAGGTAA	20	19	18347.22	467.18
98	AAAGTGATGAATCCAGCGAAACAAC	63	21	18322.99	375.15
99	AGCGAAACAACCAGCGACAGGTAAA	21	19	18321.23	408.89

100	TAAGTGAACAACCAGCTAAAGGACA	32	18	18316.08	249.56
