# Supplementary material for: An in situ-Synthesized Gene Chip for the Detection of Food-Borne Pathogens on Fresh-Cut Cantaloupe and Lettuce
Source: Front Microbiol. 2020 Feb 5;10:3089. doi: 10.3389/fmicb.2019.03089 (PMC7012807; doi:10.3389/fmicb.2019.03089)
Supplement: Supplementary file 8 [file Table_8.pdf]

Supplementary Material

Supplementary Table 8. The signal value of top 100 hybridization probe for *Vibrio parahaemolyticus*

No.	Probe Sequence (5' to 3')	Row	Column	Density (mean)	Density (st.dev.)
1	GCTACTCAAGTGTCGATACGATGAT	57	27	44549.22	417.22
2	ACGGGCAAGGCTTGGCAAAACGGCA	2	26	44412.19	610.92
3	CAGAGGCTACTCAAGTGTCGATACG	52	27	43370.74	482.28
4	TACGGGCAAGGCTTGGCAAAACGGC	1	26	42938.76	769.61
5	TACTCAAGTGTCGATACGATGATTT	59	27	42469.39	581.18
6	CTACTCAAGTGTCGATACGATGATT	58	27	41397.67	544.91
7	GGCTACTCAAGTGTCGATACGATGA	56	27	40738.00	462.65
8	ACTCAAGTGTCGATACGATGATTTT	60	27	40719.84	430.66
9	GGGCAAGGCTTGGCAAAACGGCAGT	4	26	39870.06	272.36
10	TCAAGTGTCGATACGATGATTTTAA	62	27	39841.19	528.06
11	AGAGGCTACTCAAGTGTCGATACGA	53	27	39437.56	396.57
12	TGATGACCGGGTTGTTTCCTAAGCA	26	24	38689.19	499.89
13	TCGCAGAGGCTACTCAAGTGTCGAT	49	27	38606.62	539.26
14	CAAGTGTCGATACGATGATTTTAAG	63	27	38562.77	648.72
15	AAGTCATGCCGATAATGACCATGCT	117	27	37859.16	1346.44

16	AAAACGGCAGTATAGCTGAGCAAGT	43	26	37844.30	469.72
17	TGTCGATACGATGATTTTAAGTCAT	67	27	37780.88	625.97
18	TATGATACGGGCAAGGCTTGGCAAA	108	25	37667.08	817.99
19	ATCGCAGAGGCTACTCAAGTGTCGA	48	27	37644.11	2885.67
20	TCGATACGATGATTTTAAGTCATGC	69	27	37585.73	597.27
21	TGTGATGACCGGGTTGTTTCCTAAG	24	24	37488.20	377.53
22	AGTCATGCCGATAATGACCATGCTG	118	27	37222.16	458.13
23	CGATACGATGATTTTAAGTCATGCT	70	27	36949.79	575.78
24	GGTTACTCAAGTGTCGATACGATGA	31	27	36907.09	684.87
25	TTACTCAAGTGTCGATACGATGATT	33	27	36811.19	264.62
26	CTTGATGTCGGGCATGGGTTGGCGG	26	25	36779.60	728.38
27	GATACGATGATTTTAAGTCATGCCG	103	27	36475.01	318.64
28	CGCAGAGGCTACTCAAGTGTCGATA	50	27	36055.08	634.66
29	GTCATGCCGATAATGACCATGCTGG	119	27	35936.19	541.94
30	TTGTGATGACCGGGTTGTTTCCTAA	23	24	35484.23	436.52
31	AGGCTTGGCAAAACGGCAGTATAGC	9	26	35413.19	589.26
32	GAGGTTACTCAAGTGTCGATACGAT	29	27	35345.74	502.16
33	TAGCTGAGCAAGTGATTACGCCAGT	80	26	35249.71	498.72
34	GCAAAACGGCAGTATAGCTGAGCAA	41	26	35104.34	621.71
35	GGCAAAACGGCAGTATAGCTGAGCA	40	26	35095.14	538.27
36	TTGGCAAAACGGCAGTATAGCTGAG	38	26	34966.44	620.62

37	CAAGGCTTGGCAAAACGGCAGTATA	7	26	34807.43	355.21
38	GTTTTACTCTATGATACGGGCAAGG	99	25	34784.93	564.81
39	GCTTGGCAAAACGGCAGTATAGCTG	36	26	34486.94	555.89
40	TCTATGATACGGGCAAGGCTTGGCA	106	25	34483.67	434.24
41	AAGTGTCGATACGATGATTTTAAGT	64	27	34450.77	468.16
42	TGGTGACCGGGTTGTTTCCTAAGCA	51	24	34387.54	801.27
43	GATTTTAAGTCATGCCGATAATGAC	111	27	34374.19	580.86
44	GCAGAGGCTACTCAAGTGTCGATAC	51	27	34274.25	592.85
45	CTCAAGTGTCGATACGATGATTTTA	61	27	34270.88	594.31
46	GCTTGATGTCGGGCATGGGTTGGCG	25	25	34186.63	485.17
47	TGATACGGGCAAGGCTTGGCAAAAC	126	25	34001.59	342.31
48	ATGATACGGGCAAGGCTTGGCAAAA	125	25	33979.22	392.81
49	GGCTTGGCAAAACGGCAGTATAGCT	10	26	33895.61	622.36
50	CTTGGCAAAACGGCAGTATAGCTGA	37	26	33859.31	390.40
51	AAGGCTTGGCAAAACGGCAGTATAG	8	26	33837.54	402.08
52	AGGTTACTCAAGTGTCGATACGATG	30	27	33837.26	426.58
53	TACGATGATTTTAAGTCATGCCGAT	105	27	33806.38	614.37
54	GCAGAGGTTACTCAAGTGTCGATAC	26	27	33714.95	546.84
55	TGATTTTAAGTCATGCCGATAATGA	110	27	33541.68	683.16
56	TTTAAGTCATGCCGATAATGACCAT	114	27	33486.16	903.99
57	AGCTGAGCAAGTGATTACGCCAGTA	81	26	33437.49	503.44

58	CCGCAGAGGCTACTCAAGTGTCGAT	111	26	33421.38	273.99
59	TGATGTCGGGCATGGGTTGGCGGTG	28	25	33138.23	494.50
60	TGACCGGGTTGTTTCCTAAGCAAGA	54	24	33135.60	323.33
61	TTAAGTCATGCCGATAATGACCATG	115	27	33045.43	638.11
62	ACGGCAGTATAGCTGAGCAAGTGAT	71	26	33040.56	277.95
63	CAACGGCAGTATAGCTGAGCAAGTG	69	26	32981.80	393.70
64	ACGGGCAAAGCTTGGCAAAACGGCA	27	26	32956.36	322.08
65	GATACGATGATTTTAAGTCATGCTG	71	27	32913.67	392.03
66	CCATTAGTGCCGAGTGGACATTTGT	3	23	32869.61	505.41
67	CAGAGGTTACTCAAGTGTCGATACG	27	27	32847.31	423.82
68	TGCTGATAATGACCATGCTGGCGGC	30	28	32829.37	409.86
69	CACCGCAGAGGCTACTCAAGTGTCG	109	26	32642.68	184.38
70	AATTGTTGTGATGACCGGGTTGTTT	18	24	32554.17	552.28
71	GCCGATAATGACCATGCTGGCGGCC	92	27	32507.09	611.91
72	AAGCTTGGCAAAACGGCAGTATAGC	34	26	32469.75	746.82
73	ACGATGATTTTAAGTCATGCCGATA	106	27	32358.04	686.24
74	GATGTCGGGCATGGGTTGGCGGTGC	29	25	32296.72	403.42
75	TACTCTATGATACGGGCAAGGCTTG	103	25	32159.75	1068.19
76	ACTTGATGTCGGGCATGGGTTGGCG	128	24	32142.21	516.90
77	GATACGGGCAAGGCTTGGCAAAACG	127	25	32111.44	271.87
78	AGCTTGGCAAAACGGCAGTATAGCT	35	26	32052.80	444.98

79	CGCAGAGGTTACTCAAGTGTCGATA	25	27	31969.97	433.80
80	TAAGTCATGCCGATAATGACCATGC	116	27	31947.86	601.92
81	TGGCAAAACGGCAGTATAGCTGAGC	39	26	31924.67	673.66
82	GATGATTTTAAGTCATGCCGATAAT	108	27	31902.14	511.33
83	ATACGATGATTTTAAGTCATGCCGA	104	27	31896.90	646.10
84	GTTACTCAAGTGTCGATACGATGAT	32	27	31884.83	391.30
85	TAAGACATGTTATGCCTCGTTACGT	57	23	31772.47	503.53
86	GCTCGTTTTAAGACATGTTATGCCT	49	23	31758.44	433.68
87	ATTGTTGTGATGACCGGGTTGTTTC	19	24	31744.69	784.52
88	GGCAACCCATTAGTGCCGAGTGGAC	100	22	31734.44	615.37
89	GGTGACCGGGTTGTTTCCTAAGCAA	52	24	31731.06	406.92
90	ATACGATGATTTTAAGTCATGCTGA	72	27	31719.93	414.15
91	CGGCAGTATAGCTGAGCAAGTGATT	72	26	31671.37	225.63
92	GTGCTCGTTTTAAGACATGTTATGC	47	23	31539.87	426.78
93	TTTACTCTATGATACGGGCAAGGCT	101	25	31532.05	1013.89
94	GAGTGGACATTTGTTATTGCTGTAG	14	23	31523.16	627.59
95	GGCAGTATAGCTGAGCAAGTGATTA	73	26	31390.53	210.69
96	AAAGCTTGGCAAAACGGCAGTATAG	33	26	31369.34	575.36
97	TTGATGTACTTGATGTCGGGCATGG	121	24	31302.94	525.34
98	TAACCAAACTTGGCGTATTGATGTA	104	24	31285.55	863.21
99	ACATTTGTTATTGCTGTAGTGAGTG	20	23	31244.22	507.44

100	CAAAGCTTGGCAAAACGGCAGTATA	32	26	31164.77	496.19
