# Supplementary material for: An in situ-Synthesized Gene Chip for the Detection of Food-Borne Pathogens on Fresh-Cut Cantaloupe and Lettuce
Source: Front Microbiol. 2020 Feb 5;10:3089. doi: 10.3389/fmicb.2019.03089 (PMC7012807; doi:10.3389/fmicb.2019.03089)
Supplement: Supplementary file 9 [file Table_9.pdf]

Supplementary Material

Supplementary Table 9. The signal value of top 100 hybridization probe for *E. coli* O157:H7

No.	Probe Sequence (5' to 3')	Row	Column	Density (mean)	Density (st.dev.)
1	TTTACAACGATTGCTTTATTTGGTT	7	30	20849.68	476.48
2	CGTTCTGAATTGGTGTTGCTCATTC	36	30	20116.85	314.59
3	TTACAACGATTGCTTTATTTGGTTA	8	30	19089.02	216.05
4	CGATTGCTTTATTTGGTTATCGTTC	14	30	19005.98	274.13
5	CGTTCTGAATTGGTGTTGCTCATTA	34	30	18347.65	380.72
6	TATTTTACAACGATTGCTTTATTTG	4	30	16595.95	463.28
7	GTTATCGTTCTGAATTGGTGTTGCT	29	30	16460.26	352.22
8	AACGATTGCTTTATTTGGTTATCGT	12	30	16301.73	363.61
9	TTATCGTTCTGAATTGGTGTTGCTC	30	30	16204.48	341.83
10	GTTCTGAATTGGTGTTGCTCATTAT	35	30	13787.38	536.95
11	TATATTTTACAACGATTGCTTTATT	2	30	13560.78	496.32
12	ATATTTTACAACGATTGCTTTATTT	3	30	13353.02	465.41
13	GTTCTGAATTGGTGTTGCTCATTCT	37	30	12291.31	725.04
14	ACAACGATTGCTTTATTTGGTTATC	10	30	12274.05	495.84
15	GAGCATAAATTCAAACAGAGGACCA	71	29	11981.59	215.36

16	GCATTAATTATTCTTTATGATGAGC	50	29	11592.88	411.67
17	GGTTATCGTTCTGAATTGGTGTTGC	28	30	11529.14	314.28
18	TTTTGGTAATATAGTTGTGTTTGCA	28	29	11470.70	334.53
19	GGTGTTGCTCATTCTTCAATATATA	47	30	11446.03	203.65
20	GAATTGGTGTTGCTCATTCTTCAAT	42	30	10966.81	504.25
21	CATTAATTATTCTTTATGATGAGCA	51	29	10914.76	410.57
22	GAATAGCTGAAGGTAATGGACTTTA	88	28	10825.94	245.29
23	TTTATTTGGTTATCGTTCTGAATTG	21	30	10755.55	164.51
24	TTAATTATTCTTTATGATGAGCATA	53	29	10728.12	401.09
25	ATAAATTCAAACAGAGGACCATCAT	75	29	10715.74	224.36
26	TTGGTGTTGCTCATTCTTCAATATA	45	30	10578.24	252.90
27	TAGAATAGCTGAAGGTAATGGACTT	86	28	10460.73	212.73
28	TAAATTCAAACAGAGGACCATCATA	76	29	10410.34	217.59
29	GCATAAATTCAAACAGAGGACCATC	73	29	10380.59	178.68
30	TTCTGAATTGGTGTTGCTCATTCTT	38	30	10315.24	804.70
31	TAGCTGAAGGTAATGGACTTTACAT	91	28	9902.24	221.65
32	ATAGCTGAAGGTAATGGACTTTACA	90	28	9886.67	283.18
33	ATGATGAGCATAAATTCAAACAGAG	66	29	9791.52	142.47
34	TGGTTATCGTTCTGAATTGGTGTTG	27	30	9748.99	221.02

35	TTAGAATAGCTGAAGGTAATGGACT	85	28	9592.58	225.67
36	AGCTGAAGGTAATGGACTTTACATT	92	28	9521.48	276.73
37	TGCATTAATTATTCTTTATGATGAG	49	29	9406.89	271.18
38	TGGTGTTGCTCATTCTTCAATATAT	46	30	9310.53	270.32
39	ATTTGGTTATCGTTCTGAATTGGTG	24	30	9185.75	329.64
40	AAACAGAGGACCATCATATTTGTAT	83	29	9172.11	163.00
41	TATGATGAGCATAAATTCAAACAG A	65	29	9134.78	158.10
42	CAAACAGAGGACCATCATATTTGTA	82	29	8950.68	126.36
43	GTGTTGCTCATTCTTCAATATATAT	48	30	8832.07	286.99
44	GTTTGCATTAATTATTCTTTATGAT	46	29	8796.94	320.84
45	AACAGAGGACCATCATATTTGTATA	84	29	8787.01	99.65
46	CAGAGGACCATCATATTTGTATATT	86	29	8722.06	133.18
47	TGATGAGCATAAATTCAAACAGAG G	67	29	8712.74	226.72
48	TTCAAACAGAGGACCATCATATTTG	80	29	8696.81	144.51
49	ACAGAGGACCATCATATTTGTATAT	85	29	8642.54	106.14
50	CTTTATGATGAGCATAAATTCAAAC	62	29	8594.46	182.95
51	TATTCTTTATGATGAGCATAAATTC	58	29	8514.18	325.28
52	GTAATATAGTTGTGTTTGCATTAAT	33	29	8419.33	291.92
53	TCCTAAAATAAAAAGAATAATAGG G	48	31	8332.56	163.52
54	TGGACTTTACATTAGATATATTAAA	1	29	8286.13	258.22

55	AGCATAAATTCAAACAGAGGACCA T	72	29	8097.88	277.18
56	AATTATTCTTTATGATGAGCATAAA	55	29	8083.05	180.93
57	TCAAACAGAGGACCATCATATTTGT	81	29	7921.81	274.12
58	TGGTAATATAGTTGTGTTTGCATTA	31	29	7916.40	256.82
59	GTTGTGTTTGCATTAATTATTCTTT	41	29	7857.35	447.16
60	AAACTTAGAATAGCTGAAGGTAAT G	81	28	7818.86	162.46
61	TGAATTGGTGTTGCTCATTCTTCAA	41	30	7813.98	807.36
62	ATTCTTTATGATGAGCATAAATTCA	59	29	7771.72	335.04
63	GCTGAAGGTAATGGACTTTACATTA	93	28	7733.73	215.48
64	ATTATTCTTTATGATGAGCATAAAT	56	29	7724.55	242.20
65	CCGTAATCCTAAAATAAAAAGAAT A	42	31	7702.77	297.64
66	ACCGTAATCCTAAAATAAAAAGAA T	41	31	7681.29	273.11
67	TCTTTATGATGAGCATAAATTCAAA	61	29	7679.83	166.28
68	CCTAAAATAAAAAGAATAATAGGG T	49	31	7665.13	218.05
69	AATATAGTTGTGTTTGCATTAATTA	35	29	7655.29	221.57
70	TAATATAGTTGTGTTTGCATTAATT	34	29	7653.06	190.12
71	TGTTTGCATTAATTATTCTTTATGA	45	29	7586.19	292.89
72	ATTTTACAACGATTGCTTTATTTGG	5	30	7541.52	978.81
73	CTGAATTGGTGTTGCTCATTCTTCA	40	30	7538.31	973.99

74	CTAAAATAAAAAGAATAATAGGGTA	50	31	7364.64	180.27
75	TTTGTATATTTTACAACGATTGCTT	126	29	7311.35	187.33
76	TATAGTTGTGTTTGCATTAATTATT	37	29	7306.48	330.18
77	AATAAAAAGAATAATAGGGTATTTT	54	31	7280.48	191.72
78	AAATAAAAAGAATAATAGGGTATTT	53	31	7226.23	234.85
79	TAAACTTAGAATAGCTGAAGGTAAT	80	28	7219.83	144.97
80	AACCGTAATCCTAAAATAAAAAGAA	35	31	7218.86	198.11
81	TAATTATTCTTTATGATGAGCATAA	54	29	7177.65	258.19
82	AAAATAAAAAGAATAATAGGGTATT	52	31	7030.73	161.68
83	ATAGTTGTGTTTGCATTAATTATTC	38	29	7012.73	220.40
84	TATTTTGGTAATATAGTTGTGTTTG	26	29	6997.89	375.95
85	ATATTGATTACCAATATCCTGTCAA	92	30	6996.72	168.94
86	ATAACCGTAATCCTAAAATAAAAAG	33	31	6953.13	242.14
87	TTTATGATGAGCATAAATTCAAACA	63	29	6869.26	112.23
88	TAACCGTAATCCTAAAATAAAAAGA	34	31	6856.21	272.24
89	AAAAGAATAATAGGGTATTTTTTAT	58	31	6812.67	135.50
90	GGTAATATAGTTGTGTTTGCATTAA	32	29	6798.49	322.31
91	TTATTTGGTTATCGTTCTGAATTGG	22	30	6710.37	288.10
92	CAACGATTGCTTTATTTGGTTATCG	11	30	6692.23	1055.59

93	AGTTGTGTTTGCATTAATTATTCTT	40	29	6657.13	297.97
94	ATTGGTGTTGCTCATTCTTCAATAT	44	30	6634.61	868.43
95	TTTGCATTAATTATTCTTTATGATG	47	29	6611.97	297.74
96	CATAAATTCAAACAGAGGACCATCA	74	29	6604.07	321.92
97	CTAAACTTAGAATAGCTGAAGGTAA	79	28	6558.47	144.46
98	TGTGTTTGCATTAATTATTCTTTAT	43	29	6507.71	363.39
99	GTAATGGACTTTACATTAGATATAT	100	28	6471.13	103.20
100	AAAAAGAATAATAGGGTATTTTTTA	57	31	6416.90	131.53
